# Cardiovascular and Cerebrovascular Events in the Randomized, Controlled Alzheimer's Disease Anti-Inflammatory Prevention Trial (ADAPT)

**DOI:** 10.1371/journal.pctr.0010033

**Published:** 2006-11-17

**Authors:** ADAPT Research Group

## Abstract

**Objectives:**

The Alzheimer's Disease Anti-inflammatory Prevention Trial (ADAPT) was designed to evaluate the conventional NSAID naproxen sodium and the selective COX-2 inhibitor celecoxib for primary prevention of Alzheimer's dementia (AD). On 17 December 2004, after the Adenoma Prevention with Celecoxib (APC) trial reported increased cardiovascular risks with celecoxib, the ADAPT Steering Committee suspended treatment and enrollment. This paper reports on cardiovascular and cerebrovascular events in ADAPT.

**Design:**

ADAPT is a randomized, placebo-controlled, parallel chemoprevention trial with 1–46 mo of follow-up.

**Setting:**

The trial was conducted at six field sites in the United States: Baltimore, Maryland; Boston, Massachusetts; Rochester, New York; Seattle, Washington; Sun City, Arizona; and Tampa, Florida.

**Participants:**

The 2,528 participants were aged 70 y and older with a family history of AD.

**Interventions:**

Study treatments were celecoxib (200 mg b.i.d.), naproxen sodium (220 mg b.i.d.), and placebo.

**Outcome measures:**

Outcome measures were deaths, along with nonfatal myocardial infarction (MI), stroke, congestive heart failure (CHF), transient ischemic attack (TIA), and antihypertensive treatment recorded from structured interviews at scheduled intervals. Cox proportional hazards regression was used to analyze these events individually and in several composites.

**Results:**

Counts (with 3-y incidence) of participants who experienced cardiovascular or cerebrovascular death, MI, stroke, CHF, or TIA in the celecoxib-, naproxen-, and placebo-treated groups were 28/717 (5.54%), 40/713 (8.25%), and 37/1070 (5.68%), respectively. This yielded a hazard ratio (95% confidence interval [CI]) for celecoxib of 1.10 (0.67–1.79) and for naproxen of 1.63 (1.04–2.55). Antihypertensive treatment was initiated in 160/440 (47.43%), 147/427 (45.00%), and 164/644 (34.08%). This yielded hazard ratios (CIs) of 1.56 for celecoxib (1.26–1.94) and 1.40 for naproxen (1.12–1.75).

**Conclusions:**

For celecoxib, ADAPT data do not show the same level of risk as those of the APC trial. The data for naproxen, although not definitive, are suggestive of increased cardiovascular and cerebrovascular risk.

## Editorial Commentary

**Background:** Evidence from observational studies suggests that people taking certain nonsteroidal anti-inflammatory drugs (NSAIDs) are at lower risk of developing Alzheimer's disease. However, in order to reliably find out whether NSAIDs reduce the risk of Alzheimer's, it is important to perform a properly designed randomized trial. Such a trial, ADAPT, was sponsored by the United States National Institute on Aging, and the study started recruitment in 2001. The trial involved three treatment arms: naproxen (one type of NSAID), celecoxib (another type of NSAID, but one that specifically inhibits an enzyme called COX-2), and placebo, acting as a control. It was planned that 2,625 participants would be recruited and that the primary outcome of interest was incidence of Alzheimer's disease in the three treatment arms; the trial would run for 7 y. However, this trial was terminated early, a decision based in part on information from other studies that demonstrated an increased risk of certain harms, such as heart attacks and strokes, in people taking celecoxib and other types of COX-2 inhibitors. Therefore meaningful data were not available at the time on the study's primary outcome (prevention of Alzheimer's disease). However, data about the chance of these harms are available from the ADAPT results, and these results are presented here.

**What this trial shows:** The investigators compared frequency of particular types of harm in the treatment arms: heart attack, stroke, congestive heart failure (CHF), and transient ischemic attack (TIA). For each individual type of event, some were more likely in people treated with celecoxib compared with placebo, but others were not. When considering people taking naproxen, all four types of adverse events were more likely to occur in the treatment group as compared to placebo. The investigators then combined data from all four types of harm together, and here they found that the overall risk in people taking celecoxib was higher than for people taking placebo, but that this was not statistically significant, so it could have been due to chance alone. When considering naproxen as compared with placebo, the researchers saw an approximately 60% increase in risk for all four harms combined, and this result was statistically significant. The death rate in people taking either celecoxib or naproxen was higher than for those taking placebo, but this was not statistically significant, and therefore could have been due to chance.

**Strengths and limitations:** Strengths of this study include the randomization procedures, which used a distributed computer system to assign patients to treatment arms (minimizing the chance of bias), blinding of patients to their treatment assignment, and blinding of the committee reviewing deaths and safety reports to treatment assignment. One limitation is that although the trial was large and appropriately powered for the main outcome (prevention of Alzheimer's disease), the number of safety events reported here were small and the trial was not primarily designed to examine safety. Further, participants eligible to join this trial were required to have a family history of Alzheimer's disease, so it is possible that their risk factors are slightly different from the general population.

**Contribution to the evidence:** The cardiovascular safety of NSAID's, including COX-2 inhibitors, is an intensely debated topic. Very few published data exist on the long-term safety of celecoxib as compared with placebo, although there are a number of as-yet-unpublished studies. These data on harms provided by ADAPT provide important results that should be incorporated into future meta-analyses. Such meta-analyses will give a more rigorous and reliable assessment of the safety of the drugs studied here.

## INTRODUCTION

Epidemiological studies suggest that nonsteroidal anti-inflammatory drugs (NSAIDs) may delay or prevent the onset of Alzheimer's dementia (AD). A recent meta-analysis of three prospective studies indicated a relative hazard (with 95% confidence interval [CI]) of 0.42 (0.26–0.66) among individuals with 2 y or more of sustained NSAID treatment, compared to nonusers [1]. The Alzheimer's Disease Anti-inflammatory Prevention Trial (ADAPT) was designed to investigate whether the nonselective NSAID naproxen sodium (Aleve, from Bayer, Leverkusen, Germany) or the selective cyclooxygenase-2 (COX-2) inhibitor celecoxib (Celebrex, from Pfizer, New York, New York, United States) can prevent AD or delay cognitive decline.

On Friday, 17 December 2004, increased cardiovascular risks with celecoxib were reported from the National Cancer Institute-sponsored Adenoma Prevention with Celecoxib (APC) trial. On the same day, the ADAPT Steering Committee suspended treatment with celecoxib as well as with naproxen. Suspension of naproxen reflected the ADAPT investigators' reluctance to imply, by continuing the trial, that naproxen was safer than celecoxib when ADAPT data did not support this conclusion. Our rationale for suspending both treatments in ADAPT was more fully discussed in our 18 February 2005 testimony to the Food and Drug Administration (FDA) Arthritis Advisory Committee and the Drug Safety and Risk Management Advisory Committee [2].

This report describes the structure of ADAPT and presents the trial's safety data through the end of the treatment period. We offer our interpretation of these data in their context, that is, for a trial that was not designed or powered for cardiovascular or cerebrovascular events as primary outcomes and, additionally, was stopped early. We believe we are obligated to make these results available to the scientific community and the public at large, and we present them as a contribution to the larger body of evidence about the potential cardiovascular and cerebrovascular risks of COX-2 inhibitors and conventional NSAIDs.

## METHODS

### Study Design

ADAPT is a randomized, placebo-controlled, multicenter, primary prevention trial. It is investigator-initiated and sponsored by the National Institute on Aging (NIA) via a cooperative agreement. All pertinent institutional review boards approved the study protocol.

### Participants

Recruitment was accomplished primarily through mailings to Medicare beneficiaries targeted by age and by ZIP code to areas surrounding the trial's six field sites in the United States (Baltimore, Maryland; Boston, Massachusetts; Rochester, New York; Seattle, Washington; Sun City, Arizona; and Tampa, Florida). Eligibility criteria included being the age of 70 y or older and having a history of at least one first-degree relative with Alzheimer-like dementia. Participants underwent cognitive screening to exclude those with dementia or other cognitive disorders. Persons regularly using NSAIDs were excluded. Use of aspirin was allowed at 81 mg per day or less. More specific information on eligibility criteria is available from the trial's protocol (available at http://www.jhucct.com/adapt/manall43.pdf). Written consent was obtained from each participant.

### Interventions

Participants were randomly assigned to receive celecoxib (200 mg b.i.d.), naproxen sodium (220 mg b.i.d.), or placebo.

### Randomization—Sequence Generation

The randomization sequence was generated by the trial's coordinating center in permuted blocks stratified by three age groups (ages 70–74, ages 75–79, and ages 80+) and the six field sites, with an assignment ratio of 1:1:1.5.

### Randomization—Allocation Concealment

Treatment assignment was in the form of an allocation to one of 49 bins (14 containing celecoxib and placebo matched to naproxen, 14 containing naproxen and placebo matched to celecoxib, and 21 containing placebos matched to celecoxib and naproxen). The bin system simplified long-term supply of study drug, and the number of bins reduced the risk of unmasking large numbers of participants. Only select coordinating center personnel knew the correspondence of bin allocation to treatment assignment.

### Randomization—Implementation

Randomization was implemented via computer systems distributed to each of the six sites. The sequence of bin assignments was concealed from site personnel via encoding and password protection of the randomization files. Before randomization, site personnel were required to enter baseline data. The ADAPT computer system confirmed eligibility before releasing the bin assignment.

### Masking

Masking of participants and field site personnel was achieved using a double placebo design [3]; each bin, as described above, contained both celecoxib or matching placebo, in the form of white capsules in white-labeled bottles, and naproxen or matching placebo, in the form of blue tablets in blue-labeled bottles. The placebos were provided by the manufacturers of the active drugs and were not distinguishable from them.

### Sample Size

Sample size calculations were performed using a SAS macro by Shih [4] for time-to-event outcomes. The sample size provided 80% power to detect a 30% reduction in the risk of AD over up to 7 y of follow-up, based upon parameter assumptions outlined in the trial's protocol.

### Data Collection

Study clinicians collected data through in-person interviews of participants 1 mo and 6 mo after randomization and then every 6 mo thereafter. Similarly constructed telephone interviews with participants were conducted every 6 mo in the intervals between in-person visits, starting at month 3. During these routine visits and calls, masked study personnel recorded participant reports of adverse events on data forms. When indicated, according to clinical judgment, they clarified these self-reported events with participants' primary care physicians. All fatal events also were reported in real time on death reports, and death certificates were obtained when possible.

### Outcomes

The outcomes we report here are adverse events, including occurrences of death, myocardial infarction (MI), stroke, congestive heart failure (CHF), and transient ischemic attack (TIA). Fatal events were obtained from death reports, and nonfatal events were determined from scheduled follow-up interviews. Deaths are included if they occurred on or before 17 January 2005 (1 mo after suspension of treatment) and were reported by 1 July 2005. An internal committee of three physicians, who were masked to treatment assignment, reviewed all events with details recorded on death reports to classify the fatal events. Events reported during scheduled follow-up contacts are included if they were recorded by 17 April 2005 (to allow an additional 90-d window for routine follow-up) and keyed into the ADAPT database by 1 July 2005. Composite outcomes were created in a manner similar to that of the APC trial (that is, by sequentially supplementing cardiovascular and cerebrovascular deaths with categories of nonfatal events) [5]. We combined these deaths sequentially with nonfatal MI, stroke, CHF, and TIA and for each composite outcome analyzed the time to the first event. The composite outcome of cardiovascular or cerebrovascular death, MI, or stroke also is similar to that used by the Antiplatelet Trialists Collaboration [6].

We also analyzed new occurrences of treatment for hypertension among participants not receiving treatment for hypertension at baseline. This information was obtained by participant report at routine data collection times.

### Statistical Methods

Percentages of participants experiencing the events of interest were calculated by assigned treatments. Analyses of time to first occurrences of events were performed using Cox proportional hazards regression, and hazard ratios were estimated with 95%CI and Wald *p*-values. Analyses of deaths included all participants, with censoring during follow-up of individuals with unknown vital status. Analyses of events ascertained at scheduled follow-up excluded participants with no follow-up data collection and censored those lost to follow-up. Active treatments were compared individually with placebo, but not with one another. The Kaplan-Meier method was used to graph the incidence of the composite outcome. Secondary analyses considered events occurring while participants were being issued the study drug, and events were stratified by baseline use of aspirin for cardioprotection. All analyses were conducted with SAS version 8.1.

### Data Monitoring

The trial's Treatment Effects Monitoring Committee met in person twice a year to review efficacy and safety data classified by treatment assignment. The committee, comprising four independent voting members and three nonvoting members from the trial (the NIA project officer, the director of the coordinating center, and a study consultant with special expertise in AD epidemiology), was advisory to the Steering Committee and the NIA.

## RESULTS

### Participant Flow and Recruitment

**Figure 1:**
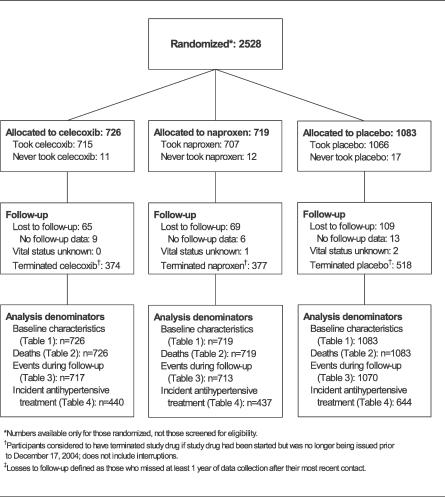
Participant Flow Diagram

The flow of participants through randomization, follow-up, and analysis is diagramed in Figure 1. Enrollment commenced in March 2001, and as of 17 December 2004, 2,528 participants had been enrolled. Of these, 98.4% received their assigned study treatment.

### Baseline Data

Table 1 provides baseline and demographic characteristics by treatment group. More men than women were enrolled. Race/ethnicity was predominantly Caucasian. Over three-quarters of the population had post-high school education. Medical history included an uncomplicated peptic ulcer or MI for small percentages of participants. Current smoking was rare. These baseline characteristics were similar for the three treatment groups, with the exception of a somewhat greater proportion of prior smokers in the placebo-treated group.

**Table 1:**
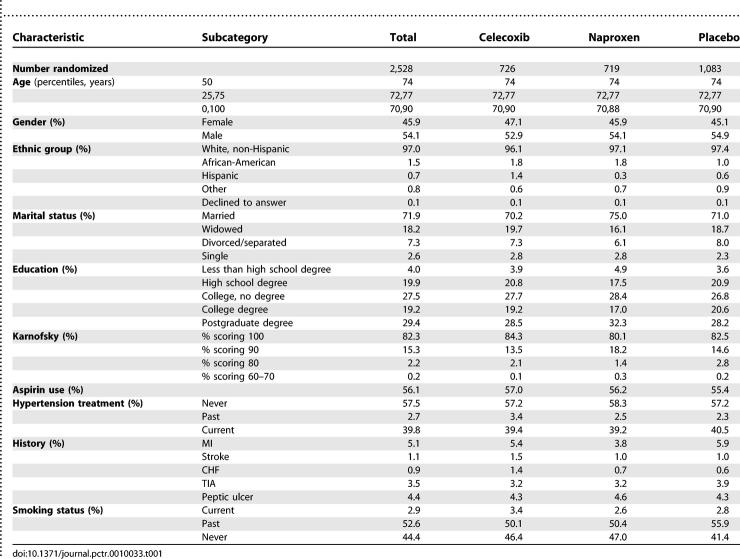
Study Population at Entry

### Numbers Analyzed, Follow-Up, and Adherence

All participants contributed to analyses of mortality. Vital status was unknown for only three participants, who could not be contacted at the time of treatment suspension or within a 6-mo window before or after that time. Of the participants, 2,500 (98.9%) had follow-up for nonfatal events. Participants contributed 4,660 person-years of follow-up through 17 January 2005 (1,346 for celecoxib, 1,332 for naproxen, and 1,982 for placebo). The median follow-up times for the three treatment groups were 23.3, 23.5, and 22.1 mo, respectively. Median times of administration of the study drug were 16.1, 14.0, and 15.6 mo, respectively (*p* = 0.32).

### Outcomes and Estimation

**Figure 2:**
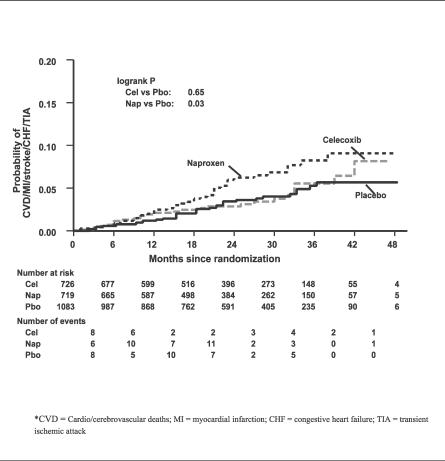
Graph of Time to Cardiovascular Death, MI, Stroke, CHF, or TIA

Table 2 presents deaths (counts and cumulative 3-y incidence) by treatment group through 17 January 2005. Mortality was low, considering participant ages. The hazard ratio point estimates for the active treatments compared to placebo exceeded 1.00 for overall mortality and for cardiovascular and cerebrovascular deaths, but the corresponding 95% CIs were wide. Table 3 shows the hazard ratios for fatal and nonfatal MI, stroke, CHF, and TIA and for our sequential composite outcomes, and Figure 2 is a Kaplan-Meier graph of the cumulative incidence over time for the full composite outcome. For celecoxib, the hazard ratios exceeded 1.00 for two of the four individual events, and the composite outcomes were only slightly elevated, none of them at a level of conventional statistical significance. For naproxen, the hazard ratios were elevated for each individual cardiovascular and cerebrovascular event, again with wide CIs. As these event categories were sequentially combined with cardiovascular and cerebrovascular deaths into composite outcomes, the hazard ratios converged around a point estimate of an approximate 60% increase in risk, and the boundary of conventional statistical significance was crossed.

**Table 2:**
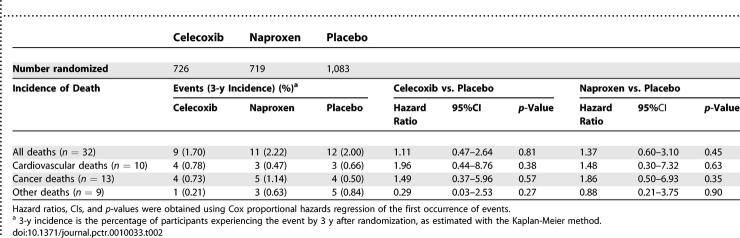
Incidences and Hazard Ratios for Deaths by Treatment Group

**Table 3:**
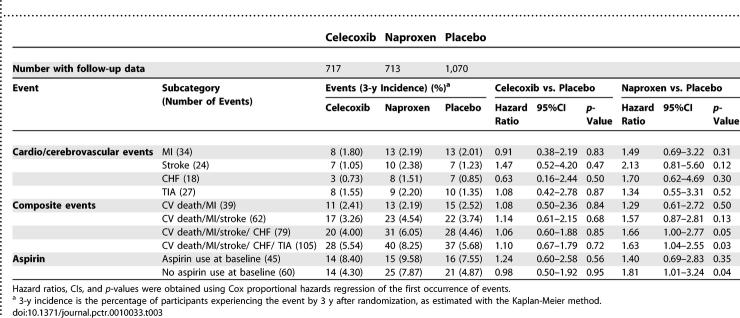
Incidences and Hazard Ratios for First Occurence of Events by Treatment Group

### Ancillary Analyses

Hazard ratios were similar for secondary analyses that considered only the time during which participants were administered study treatments. Analyses stratified for baseline use of aspirin showed the highest risk among those in the naproxen group who were not taking aspirin, but the tests of interaction by aspirin use were not significant for either drug (*p* = 0.56 for celecoxib and *p* = 0.59 for naproxen).

Of the 1,521 participants who reported that they were not receiving treatment for hypertension at baseline, 471 newly reported receiving treatment for hypertension during follow-up. Table 4 shows the counts (with cumulative 3-y incidence) for the groups assigned to celecoxib, naproxen, and placebo. Both study treatments were associated with a substantial and highly significant increase in newly reported treatment for hypertension.

**Table 4:**
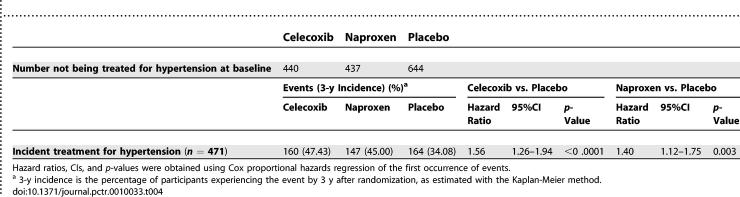
Incidences and Hazard Ratios for Initiation of Hypertension Therapy by Treatment Group

## DISCUSSION

### Interpretation

For celecoxib, the ADAPT safety results showed no consistent tendency toward increased or decreased risk for cardiovascular and cerebrovascular events. Composite outcomes showed only slightly increased estimates that were not statistically significant but, if true, would be clinically significant when applied to the large population of celecoxib users. The ADAPT naproxen results showed moderately elevated risks for all four cardiovascular and cerebrovascular events analyzed, and the CIs for the composite outcomes narrowed as additional events were included. Both drugs were associated with increased indication for antihypertensive treatment.

As noted above, these results must be interpreted in the context of a trial that was stopped early, with small numbers of events that were not originally intended as specific outcomes. Therefore, our intention is not to present the ADAPT data as being conclusive, but rather to make them available to the general medical and research community and to the public at large as a contribution to the growing body of knowledge from placebo-controlled trials of COX-2 inhibitors and to the more sparse knowledge from placebo-controlled trials regarding conventional NSAIDs, as reviewed below.

### Generalizability

ADAPT participants are a select population by virtue of their family history of Alzheimer-like dementia, their high educational achievement, and their relatively healthy status, as evidenced by the low rates of cardiovascular disease (CVD) at entry and the low mortality rate observed during the trial. However, these factors limit generalizability only to the extent that they modify the treatment effect. There has been speculation that any increased cardiovascular risk from anti-inflammatory drugs may be confined to those at higher risk of disease, but there is little rationale for or evidence of differing response to anti-inflammatory treatment.

### Overall Evidence

The cardiovascular safety of COX-2 inhibitors has been questioned since the publication of the Vioxx Gastrointestinal Outcomes Research (VIGOR) trial results in late 2000, in which rofecoxib (Vioxx) was associated with a 5-fold increased risk of MI [7]. The Celecoxib Long-term Arthritis Safety Study (CLASS) did not show an elevated cardiovascular risk with celecoxib use [8], but CLASS differed from VIGOR in that use of low-dose aspirin for cardioprotection was allowed (as in ADAPT), and the comparator treatments included ibuprofen, a compound later thought to mitigate the cardioprotective effects of aspirin [9,10]. A class effect of COX-2 inhibiting drugs was subsequently suggested [11,12]. The absolute annualized rates of cardiovascular events in VIGOR and CLASS were similar, and both rates were higher than those obtained from pooling the placebo groups from several large primary prevention trials [12]. Nevertheless, a 2003 meta-analysis of all clinical trials with celecoxib did not show increased cardiovascular risk compared to NSAID use or placebo (though only six trials of 4- to12-wk duration were placebo controlled, resulting in just 200 person-years of exposure to placebo and three events) [13]. Also, a large observational study in a Kaiser Permanente database showed decreased odds of MI with celecoxib, but a 3-fold increased odds with use of rofecoxib compared to remote use of NSAIDs [14].

On 30 September 2004 Merck withdrew rofecoxib from the market, after results from the Adenomatous Polyp Prevention on Vioxx (APPROVe) trial showed a statistically significant relative risk for thrombotic events of 1.92 for rofecoxib compared with placebo [15]. On 15 October 2004 Pfizer announced that its drug valdecoxib (Bextra) appeared to increase the risk of cardiovascular events when given following coronary artery bypass grafting (relative risk = 3.7, as later published) [16]. The debate continued as to whether the cardiovascular risks observed were drug specific or represented a class effect of COX-2 inhibitors [17,18]. Then, on 17 December 2004, the APC trial announced its early termination because of observed hazard ratios for cardiovascular death, MI, stroke, or heart failure of 2.3 with 200 mg b.i.d. celecoxib and 3.4 with 400 mg b.i.d. celecoxib, compared to placebo [5]. Although the Pfizer-sponsored Prevention of Sporadic Adenomatous Polyps (PreSAP) trial was reported not to have shown increased risk with celecoxib use, it too was terminated. At that point, the ADAPT Steering Committee, in consultation with the NIA and the chair of the Treatment Effects Monitoring Committee, voted to suspend treatment with celecoxib because the same dosage produced notable cardiovascular risk in the APC trial. The FDA put a full clinical hold on prevention trials with celecoxib 6 d later.

As of the writing of this manuscript, the APC paper [5] is the only peer-reviewed publication specifically reporting on cardiovascular events from a placebo-controlled trial of celecoxib; results from the PreSAP trial, including cardiovascular events, have recently been published as well [19]. A meta-analysis of COX-2 inhibitor trials, using data supplied by the drug manufacturers or presented at the February 2005 FDA Joint Advisory Committee meeting, was recently published [20]. The long-term placebo-controlled celecoxib data were largely attributable to the APC and PreSAP trials. However, other long-term placebo-controlled trials of celecoxib have been conducted. A systematic search of ClinicalTrials.gov, MEDLINE, the Cochrane Library and CENTRAL registry, and clinicalstudyresults.org identified 38 placebo-controlled trials of celecoxib of 12 wk or longer duration. At least 14 of these (of which ADAPT is one) appear to have sample sizes of 100 or more and median treatment duration of 1 y or more. A meta-analysis of such trials may lead to a more precise estimate of the long-term risks of the medication. A National Institutes of Health-led effort to obtain cardiovascular and cerebrovascular outcome data from such trials and to conduct a meta-analysis is underway.

While the ADAPT celecoxib data may be compared with corresponding results from sister trials of similar size and duration, our naproxen data stand alone. Several trials have reported that naproxen produced lower cardiovascular risks than comparator COX-2 inhibitor treatments, and the meta-analysis by Kearney et al. [20] estimates the risk ratio for COX-2 inhibitors compared to naproxen (500 mg b.i.d.) at 1.57. However, these results do not answer the question of risk with naproxen use.

It has been suggested that naproxen, at least at higher doses, produces lower cardiovascular risk than comparator COX-2 drugs because of antithrombotic effects similar to those of aspirin. Numerous observational studies have investigated the association of naproxen use and CVD, and a meta-analysis of eight case-control studies and three retrospective cohort studies obtained a combined estimate of 0.86 [21]. However, as was noted by FDA drug safety researcher David Graham in his testimony on 17 February 2005 before the FDA Arthritis Advisory Committee and the Drug Safety and Risk Management Advisory Committee [22], the three cohort studies did not show a significant protective effect. Also, among the three case-control studies showing the largest risk reduction, one compared naproxen use to other NSAID use (primarily ibuprofen) rather than to no use or remote use [23], another included subarachnoid hemorrhage and subdural hematoma in a composite outcome [24], and the third showed a similar cardioprotective effect for both remote and current use of naproxen [25]. More recently, several nested or population-based case-control studies have shown moderately increased cardiovascular risk associated with the use of naproxen [14,26,27]. Of course, these are all observational studies that are subject to potential biases in the use and selection of NSAID therapy.

Several published trials tested naproxen and placebo as well as a COX-2 inhibitor, and three reported on the outcome of incident hypertension [28–30]. One of these trials had a sample size of 351 [28], and the other two had follow-up times of 12 wk and 26 wk [29,30]. Though the numbers of events were small, all three showed more hypertension with naproxen than with placebo, consistent with results from ADAPT. The meta-analysis by Kearney et al. [20] provides a risk ratio estimate for naproxen compared to placebo of 0.92. However, this estimate is obtained from trials with treatment durations of 12 wk or less, with the exception of two of the trials discussed above [28,30].

The ADAPT data on naproxen are by no means definitive. Under ordinary circumstances, they would not have warranted suspension of treatment in December 2004. Nonetheless, after the APC announcements, the ADAPT investigators were fundamentally uncomfortable with continuing naproxen simply because highly publicized external circumstances had spotlighted risks with celecoxib, but not with naproxen. It has been suggested that all NSAIDs may have some degree of cardiovascular risk [22,26,31], perhaps especially for those not taking aspirin. The ADAPT naproxen data provide some support to this theory, particularly for older adults. However, without adequate evidence from long-term, placebo-controlled trials, the measure of this risk remains a matter of speculation.

There are several limitations in the ADAPT data. First, because ADAPT was not designed to detect differences in cardiovascular and cerebrovascular risks, the composite outcomes were specified post hoc. We designed their constituent events both to be as comparable as possible to the approaches used in the Antiplatelet Trialists Collaboration and the APC trials and to draw on outcomes collected in a systematic manner from close-ended questions asked to participants at every follow-up contact. Because ADAPT by design focused on neurological events, we asked participants specifically about the occurrence of both stroke and TIA. Although we also asked about MI and CHF, we did not ask specific questions about conditions such as unstable angina or other indications for interventional cardiologic procedures and therefore were not able to include them in our composite of systematically collected outcomes.

We also did not have a priori procedures for adjudication of cardiovascular or cerebrovascular events. Although we could employ such procedures post hoc in an attempt to increase the precision of reported events, these procedures have several limitations. We cannot identify missed events, and we cannot be certain that the process of adjudication is not influenced by the current controversy. Therefore, adjudication will only decrease the absolute risk observed and could bias the relative risk. Also, adjudication may foster a sense of precision that is inappropriate given the small numbers of reported events in a trial that was terminated early [32]. For these reasons, we have chosen to present what we believe to be unbiased raw data that provide a valid estimate of both the absolute and relative treatment effects.

Ultimately, the largest limitation in the ADAPT data is the imprecision resulting from the small numbers of events. A corresponding caution in their interpretation is warranted. Even so, our actions, as well as those of other investigators and the FDA, reflect the low tolerance for risk in primary prevention trials. For their participants, the only benefit of the intervention is the possibility of later protection from a condition they might have avoided in any case. This creates some difficulty for extrapolation to the clinical setting, where these drugs are used for pain relief in conditions such as osteoarthritis and rheumatoid arthritis, and the potential risks need to be weighed against the known immediate benefits of treatment. Nevertheless, we as a medical and research community are attempting just such difficult extrapolation, as the primary prevention setting is providing long-term, placebo-controlled data for drugs approved on the basis of short-term trials but used for the treatment of chronic conditions frequently necessitating decades of analgesic therapy. Therefore, we believe it is essential to make data from long-term trials such as ADAPT available to contribute to the larger body of evidence on drug safety.

## SUPPORTING INFORMATION

CONSORT Checklist Click here for additional data file. (47 KB DOC)

Trial Protocol: ADAPT Protocol, Version 1.4 Click here for additional data file. (233 KB PDF)

## COMPETING INTERESTS

The writing committee that takes primary responsibility for this paper (see Acknowledgments) reports the following competing interests. Barbara Martin, Denis Evans, and Curtis L. Meinert have no conflicts of interest to report. John C. S. Breitner previously held partial royalty interests in two United States patents for the use of NSAIDs for the prevention of Alzheimer's disease; in January 2005 he assigned these interests irrevocably as a gift to a private charitable foundation in which he has no constructive or beneficial interest. Constantine G. Lyketsos is a consultant to GlaxoSmithKline and receives lecture honoraria and support for continuing medical education activities from AstraZeneca, Eisai, Forest Laboratories, Janssen, Lilly, Novaris, and Pfizer.

Along with the competing interests listed by the writing committee, the Steering Committee's competing interests are as follows. Drs. Mullan and Piantadosi have no conflicts of interest to report. Dr. Green has received lecture honoraria and support for continuing medical education activities from Eisai, Forest Laboratories, and Pfizer and is an unpaid scientific advisor to Myriad Pharmaceuticals. Dr. Tariot is a consultant to Abbott Laboratories, AstraZeneca, Bristol-Meyers Squibb, Eisai, Forest Laboratories, Janssen, Lilly, Myriad Pharmaceuticals, Pfizer, Sanofi, and Schwabe; he has received research support from Abbott Laboratories, AstraZeneca, Elan, Pfizer, Bristol-Myers Squibb, Forest Laboratories, Merck, Neurochem, Ono, and Sanofi and lecture honoraria from Pfizer, Forest Laboratories, AstraZeneca, and Eisai. Dr. Craft is a consultant for and receives research support from GlaxoSmithKline. Dr. Sabbagh is a speaker for Pfizer, Eisai, Ortho-McNeil, Forest Laboratories, and Abbott Laboratories and receives research support from GlaxoSmithKline, Pfizer, Eisai, Elan, Ono, and Takeda. Dr. Brandt receives royalties from Psychological Assessment Resources, Inc., publisher of the Hopkins Verbal Learning Test—Revised, one of the neuropsychological tests used in this trial; however, Dr. Brandt waived royalties from the use of the test in ADAPT. Dr. Welsh-Bohmer, who is a non-voting member of the Steering Committee, continues to hold royalty interests in these two patents; she also is a paid consultant to GlaxoSmithKline and has received speaking honoraria from Pfizer and private gifts to the Ryan ADRC from GlaxoSmithKline and Novartis.

## Abbreviations

AD: Alzheimer's dementia
ADAPT: Alzheimer's Disease Anti-inflammatory Prevention Trial
APC: Adenoma Prevention with Celecoxib trial
APPROVe: Adenomatous Polyp Prevention on Vioxx trial
b.i.d.: bis in die (twice daily)
CHF: congestive heart failure
CI: confidence interval
CLASS: Celecoxib Long-term Arthritis Safety Study
COX-2: cyclooxygenase-2
CV: cardiovascular
CVD: cardiovascular disease
FDA: Food and Drug Administration
MI: myocardial infarction
NIA: National Institute on Aging
NSAID: nonsteroidal anti-inflammatory drug
PreSAP: Prevention of Sporadic Adenomatous Polyps trial
TIA: transient ischemic attack
VIGOR: Vioxx Gastrointestinal Outcomes Research trial
