# Supplementary material for: Cardiovascular and Cerebrovascular Events in the Randomized, Controlled Alzheimer's Disease Anti-Inflammatory Prevention Trial (ADAPT)
Source: PLoS Clin Trials. 2006 Nov 17;1(7):e33. doi: 10.1371/journal.pctr.0010033 (PMC1851724; doi:10.1371/journal.pctr.0010033)
Supplement: Trial Protocol: ADAPT Protocol, Version 1.4 [file pctr.0010033.sd002.pdf]

Alzheimer's Disease Anti-inflammatory Prevention Trial

ADAPT Protocol
Version 1.4

19 Nov 2002

Document distribution

Version	Version date	Distribution	Distribution Date
0.0 (draft)	21 Jul 2000	ADAPT Steering Committee	25 Jul 2000
1.0	08 Aug 2000	Pharmacia, Bayer, Matthews Media Group, Covance, McKesson, ProClinical	08 Aug 2000
1.1	09 Aug 2000	JHU Committee on Human Research<br>FDA (with IND application) and field sites for IRB submission	09 Aug 2000<br>21 Aug 2000
		Treatment Effects Monitoring Committee	12 Sep 2000
1.2	16 Jan 2001	ADAPT Steering Committee, JHU Committee on Human Research, field sites for IRB submission	17 Jan 2001
1.3	18 Mar 2002	ADAPT Steering Committee, JHU Committee on Human Research, field sites for IRB submission	19 Mar 2002
1.3	18 Mar 2002	ADAPT Steering Committee, JHU Committee on Human Research, field sites for IRB submission<br>(updated pages: i; 63-65 only)	10 Apr 2002
1.4	19 Nov 2002	ADAPT Steering Committee, JHU Committee on Human Research, field sites for IRB submission	20 Nov 2002

Document history

Version 0.0 (21 July 2000)

Version 1.0 (08 August 2000)

Numerous editorial and wording changes were made throughout the document to improve clarity

Substantive modifications were made to the following sections:

§3.3 Inclusion / exclusion criteria:

- "Ability to speak, read, and understand English" was changed to
- "Sufficient fluency in written and spoken English to participate in study visits and neuropsychological testing"
- "Willingness to avoid use of the following for the duration of the study" was changed to "Willingness to limit use of the following for the duration of the study"
- "...vitamin E (at doses ≥ 400 IU per day)" was changed to "vitamin E (at doses > 400 IU per day)"
- "Provision of informed consent" was added as an inclusion criterion"
- "Any plans to leave site area in the next three years" was deleted as an exclusion criterion"

§4.3 Management of side effects and adverse events:

- "At the enrollment visit and all subsequent visits, study personnel will review with participants the symptoms of known potential adverse effects of the study medications. Participants will be encouraged to report these and any other side effects" was added
- "Safety reports also will be submitted to the FDA within 10 working days, as required" was added

§4.4

- "...vitamin E (at doses ≥ 400 IU per day)" was changed to "vitamin E (at doses > 400 IU per day)"

§5.4 Eligibility evaluation visit:

- "Digit span, Generative Verbal Fluency, Narratives from the Rivermead Behavioral Memory Test, Brief Visuospatial Memory Test, Self-rating of Memory Functions" were deleted from the list of neuropsychological tests administered to prospective participants"
- "Draw blood for APOE determination and/or DNA banking" was added to this visit and deleted from the enrollment visit procedures (see below)
- "Discuss the consent for DNA testing and banking" was added to this visit and deleted from the enrollment visit procedures (see below)

§5.5 Enrollment visit
- "Draw blood for APOE determination and/or DNA banking" was deleted
- "Discuss the consent for DNA testing and banking" was deleted

§6.1 Overview of followup visits
- "Target dates for followup visits are calculated from the date of enrollment. The time intervals around visit target dates are referred to as visit windows. Visit windows are contiguous (i.e., there is always an open visit window, because as one visit window closes, the subsequent one opens). The closing of one visit window and the opening of another will occur at the midpoint between the target dates of two consecutive visits" was added

§9. Treatment effects monitoring
- "In addition, a summary report will be provided to the study's parent institutional review board (IRB), The Johns Hopkins School of Hygiene and Public Health Committee on Human Research (CHR). The CHR will provide guidance regarding submission of the summary report to the participating institution's IRBs" was added
- "It is anticipated that the Steering Committee will approve the recommendations and then communicate them to the appropriate entities. In the event that the Steering Committee rejects the TEMC recommendations, the Steering Committee will follow the procedures outlined in the ADAPT policy statement on treatment effects monitoring" was added

§10.4. Section 10.4, "Certification of staff," was added

§11. Section 11 was changed from "Regulatory and ethical considerations" to "Investigational New Drug application"
- Section 11.2 was moved to section 12.1
- Section 11.3 was moved to section 12.5
- Section 11.4 was moved to section 12.2

§12. Section 12, "Protection of Human Subjects", was added
- Section 12.3, "Benefits and risks to participants," was added
- Section 12.4, "Safety monitoring," was added
- Section 12.6, "Biohazards," was added

Version 1.1 (09 August 2000)

§8.2. Analysis of primary outcome description changed from “Cox proportional hazards regression” to “descrete time methods”

§13. Section 12.6, “Biohazards,” was moved from section 12.6 to section 13

Version 1.2 (16 Jan 2001)

Editorial and wording changes were made throughout the document to improve clarity

Substantive modifications were made to the following sections:

§3.3 Eligibility criteria

- “Clinically significant liver or kidney disease” was changed to “Clinically significant hypertension, anemia, liver disease or kidney disease according to guidelines provided in the ADAPT handbook”
- “Current blood pressure ≥160 mm Hg systolic or ≥ 100 mm Hg diastolic” was deleted as an exclusion criterion
- “Current plasma potassium ≥ 5.4 mEq/L” was deleted as an exclusion criterion
- “Current hematocrit ≥ 34% (females) or ≤ 37% (males)” was deleted as an exclusion criterion
- “Use of ≥ 4 doses per week of any of the following in the 28 days prior to the eligibility evaluation visit:” was changed to “Use of ≥ 4 doses per week of any of the following in the 28 days prior to the enrollment visit:”
- “Participation in any other prevention trial for neurodegenerative disease or in any other trial (treatment or prevention), using agents or procedures likely to affect ADAPT outcomes or their assessment” was changed to “Enrollment in any trial that is likely to interfere with ADAPT procedures or affect treatment outcomes.”

§4.3 Management and reporting of side effects and adverse events

- “Serious adverse events must be reported to the Coordinating Center within 24 hours of the field site personnel learning of the event” was changed to “Serious or unexpected adverse events must be reported to the Coordinating Center within 1 working day of the field site personnel learning of the event”

§4.4 Criteria for study treatment termination or interruption

- “Participation in any other prevention trial for neurodegenerative disease or in any other trial (treatment or prevention), using agents or procedures likely to affect ADAPT outcomes or their assessment” was changed to “The participant enrolls in any trial that is likely to interfere with ADAPT procedures or affect treatment outcomes.”

- “Any condition that, in the opinion of the study physician, makes it medically inappropriate or risky for the participant to continue on study treatment” was added.

§5.3 Screening
- “Attendance by a collateral respondent is encouraged for all visits; however it will be required for the eligibility evaluation visit, the enrollment visit, all cognitive assessment visits, and dementia evaluation visits if needed” was changed to “Attendance by a collateral respondent is encouraged for all visits; however, it will be required for the eligibility evaluation visit and any dementia evaluation visits”

§5.4 Eligibility evaluation visit
- “Discuss the consent for eligibility evaluation and the consent for DNA testing and banking and ask the prospective participant to sign” was changed to “Discuss the consent for eligibility evaluation and ask the prospective participant to sign”
- “Prospective participants whose cognitive status is ambiguous may be referred for a dementia evaluation visit that will follow the same protocol to be used for later evaluations for incident dementia.” was changed to “Prospective participants whose cognitive status does not meet the study entry criteria may be referred for evaluation outside the study.”
- “Referred individuals will be invited back for a enrollment visit if they are not demented and do not show other clinically significant cognitive deficit according to an evaluation of their history, examination and test results.” was deleted
- “Draw blood for APOE determination and/or DNA banking” was moved to section 5.5

§5.5 Enrollment visit
- “Discuss the consent for enrollment and ask participant to sign” was changed to “ Discuss the consent for enrollment and the consent for DNA testing and banking and ask participant to sign”
- “Geriatric Depression Scale” was added

§6.1 Overview of followup visits
- “Ideally, visits are to be scheduled within 2 weeks of the target dates.” was added

§6.2 Cognitive assessment visits
- “Geriatric Depression Scale” was added

§7.1 Description of neuropsychological tests
- The following section was added:

"**Eligibility evaluation battery**

The eligibility evaluation battery includes two paper-and-pencil cognitive tests and 1 questionnaire administered to the participant's collateral respondent. A trained and certified psychometrician administers each of the tests according to the guidelines provided in the Eligibility Evaluation Battery and the ADAPT Neuropsychology Manual.

Cognitive tests

- *Modified Mini-Mental State (3MS)*: The measurement of global cognitive function, including orientation, language, verbal recall, recognition, long-term memory, praxis, and constructional ability, relies mainly on an adaptation of the 100-point 3MS[59]. The original test or one of the two equivalent alternate versions developed for the Cache County Study on Memory and Aging[53] is used.

- *Hopkins Verbal Learning Test - Revised (HVLT-R)*: One of the six equivalent alternate forms of the HVLT-R[60] is used to measure verbal learning and delayed recall. In this test, a list of twelve words - four from each of three semantic categories - is read to participants. Three learning trials are followed, after 20 to 25 minutes, by a delayed recall trial.

Collateral respondent questionnaire

- *Dementia Severity Rating Scale (DSRS)*: The informant version of the DSRS[61] is administered to the collateral respondent to assess cognitive and functional impairment."
- "Participants are asked to name as many animals as possible in one minute." was changed to "Participants are asked to name as many supermarket items as possible in one minute."
- "Memory scale" was replaced by "Self-administered questionnaires"
- "*Geriatric Depression Scale (GDS)*: To determine the participant's perception of depression, the 30-item GDS[66] is administered." was added

§9. Treatment effects monitoring

- "In addition, a summary report will be provided to the study's parent IRB, The Johns Hopkins School of Hygiene and Public Health Committee on Human Research (CHR)." was changed to "In addition, a summary report will be provided to the study IRBs."
- "The CHR will provide guidance regarding submission of the summary report to the participating institution's IRBs." was deleted

§11. Investigational new drug application

- "An application is being submitted by the ADAPT Chair to the FDA to request an Investigational New Drug (IND) authorization. It is therefore anticipated that the trial will be conducted under a sponsor-investigator initiated IND, to be held by the ADAPT Chair. The field site directors, associate directors, coordinators, neuropsychologists, and all study physicians will complete investigator statements (FDA form 1572) and submit them to the Coordinating Center prior to the start of the trial. The 1572 forms and the ADAPT protocol will be submitted to the FDA by the Coordinating Center before the start of enrollment." was deleted
- "The trial is being conducted under Investigational New Drug (IND) number 60,739 which is held by the ADAPT Chair." was added

§12.2 Consent process

- "The consent for eligibility evaluation and the consent for DNA testing and banking are signed at the first visit (eligibility evaluation visit) at the ADAPT field site" was changed to "The consent for eligibility evaluation is signed at the first visit (eligibility evaluation visit)"
- "At the fist visit, the consent forms for enrollment is reviewed with the participant. The participant is given an unsigned copy of the consent form to take home." was changed to "In addition, the consent form for enrollment and the consent for DNA testing and banking are reviewed with the participant at the eligibility evaluation visit. The participant at the eligibility evaluation visit, the participant is given unsigned copies of the consent forms to take home. The forms are then reviewed again with the participant and signed at the enrollment visit."

Version 1.3 (19 Mar 2002)

Editorial and wording changes were made throughout the document to improve clarity

Substantive modifications were made to the following sections:

§3.3 Inclusion criteria

- “Age 70 years or older at time of randomization” was changed to “Age 70 years or older at time of the eligibility evaluation visit”
- “Willingness to limit use of the following for the duration of the study:
  - “vitamin E (at doses > 400 IU per day)” was changed to “vitamin E (at doses >600 IU per day)”
  - “non-aspirin NSAIDs **or** aspirin (>81 mg per day)” was added;
  - “corticosteroids” was deleted;
  - “non-aspirin NSAIDs” was deleted
  - “anti-inflammatory or analgesic doses of aspirin (>81 mg per day)” was deleted

§3.3 Exclusion criteria

- “Concurrent use of systemic corticosteroids” was added
- “Use of > 4 doses per week of any of the following in the 28 days prior to the enrollment visit:
  - “vitamin E (at doses > 400 IU per day)” was changed to “ vitamin E (at doses > 600 IU per day)”;
  - “non-aspirin NSAIDs **or** aspirin (> 81 mg per day) was added;
  - “corticosteroids” was deleted
  - “non-aspirin NSAIDs” was deleted
  - “anti-inflammatory or analgesic doses of aspirin doses (> 81 mg per day)”was deleted

§4.3 Management and reporting of side effects and adverse events

- “Serious or unexpected adverse events must be reported to the Coordinating Center within 1 working day of the field site personnel learning of the event” was changed to “Serious adverse events must be reported to the Coordinating Center within 1 working day of the field site personnel learning of the event”

§4.4 Criteria for study treatment termination

- “The participant enrolls in any trial that is likely to interfere with ADAPT procedures or affect treatment outcomes” was deleted

§4.4 Criteria for study treatment interruption

- “If the participant requires corticosteroids, or warfarin, ticlopidine or any type of anti-coagulant, study treatment is interrupted for the duration of usage.” was changed to “If the participant requires systemic corticosteroids, or warfarin, ticlopidine or any type of anti-coagulant, study treatment is interrupted for the duration of usage.”

- "If the participant is taking ≥ 4 doses per week...",
  - "vitamin E (at doses > 400 IU per day" was changed to "vitamin E (at doses > 600 IU per day";
  - "non-aspirin NSAIDs **or** aspirin (>81 mg per day)" was added
  - "non-aspirin NSAIDs" was deleted
  - "anti-inflammatory or analgesic doses of aspirin (> 81 mg per day)" was deleted
- "The participant enrolls in any trial that is likely to interfere with ADAPT procedures or affect treatment outcomes" was added

§5.3 Screening

- "Attendance by a collateral respondent is encouraged for all visits; however, it will be required for the eligibility evaluation visit and any dementia evaluation visits" was changed to "Attendance by a collateral respondent is encouraged for all visits; however, it will be required for the eligibility evaluation visit"

§5.4 Eligibility evaluation visit

- "Record current medications" was changed to "Record current use of proscribed and limited-use medications"

§7.2 Diagnosis of dementia

- Section was extensively rewritten; substantive changes include the following:
  - The field site clinical team, rather than the Adjudication Committee, will be responsible for the diagnosis of dementia, including the identification of incident cases of Alzheimer's disease; a participant should be considered to have a diagnosis of dementia only when that diagnosis is considered to be reasonably certain and irreversible
  - Participants with mild cognitive impairment, or "cognitive syndrome with insufficient severity or attendant functional disability to qualify definitively as dementia" as previously described, will return to regular followup rather than returning in 3 months for a repeat dementia evaluation visit
  - Discussion of criteria for the differential diagnosis of dementia was deleted; discussion is provided in the ADAPT Handbook instead

§7.3 Followup of participants diagnosed with dementia

- Section added: "When the field site clinical team has reached a reasonably certain diagnosis of irreversible dementia, they should direct the participant to stop study treatment and should refer him or her to a dementia clinic for care. For as long as possible, however, the participant should return for regular ADAPT followup and annual cognitive assessment. When individuals with dementia are considered unable to make decisions about their continued participation in the trial, consent for such

participation must be obtained from a responsible third party, along with assent from the participant if this is meaningful."

§7.4 Incidence of Alzheimer's disease

- Section added, substantive points are:
  - Differential diagnoses are to be made by the field sites using NINCDS/ADRDA criteria
  - If a participant's status changes, the person may be re-evaluated, but the diagnosis will not be changed

§7.5 Diagnostic Review Committee

- Section added; substantive points are:
  - Diagnostic Review Committee replaces Adjudication Committee
  - Purpose of the Committee is to promote the homogeneity and standardization of diagnoses across the ADAPT field sites

§8.2 "Analysis of primary outcome" changed to "Analysis of design variable"

- "In the principal analysis, discrete time methods will be used to model the risk of developing AD while controlling for age at entry and other prospective baseline risk factors." was replaced by "In the principal analysis, time-to-event methods will be used to model the effect of assigned treatment on the development of AD. (Secondary analyses of the treatment effects will include adjustments for adherence to the assigned study treatment.)"
- Paragraph added: "The survival analysis models will control for age at entry and other prospective baseline risk factors. Because age is a strong determinate of risk for AD, and because the association of age and AD is not linear, various models that adjust for age may be created. At the least, age will be included in the same categories used for stratification at randomization (ages 70-74, 75-79, and 80+). Finer categories may be used if there is evidence of residual confounding from imbalance in the age distribution among the treatment groups. However, it is expected that with stratification and a planned sample size of 2,625, such confounding is unlikely. Age-by-treatment interactions also will be investigated using the above stratification categories."
- Paragraph added: "Other covariates to be included in the survival analysis models will include a core set of established predictors of AD incidence, as determined from epidemiological studies. Such factors include *APOE* genotype, education, gender, use of estrogen, use of anti-oxidants, and a history of head injury (family history of dementia is irrelevant here, as it is a criterion for enrollment). Again, it is expected that these variables will be balanced among treatment groups at enrollment, but analyses will be done to assess whether observed treatment effects are independent of them. Other baseline variables may be included if they are unbalanced across treatment groups."

§9. Treatment effects monitoring

- "Stopping rules may be used as guidelines in the decision-making process" was changed to "Stopping guidelines may be used in the decision-making process"

§12.1 Monitoring of IRB approval process

- "...all serious or unexpected adverse events, regardless of presumed relationship to the experimental treatment, will be reported to the parent IRB as well as to the field site IRBs." was changed to "...all serious adverse events, regardless of presumed relationship to the experimental treatment, will be reported to the parent IRB as well as to the field site IRBs."

§12.3 Risks and potential benefits to participants

- "It also is possible that participants receiving active study treatment could benefit from a lower risk of heart attack, stroke, and certain cancers" was changed to "It also is possible that participants receiving naproxen could benefit from a lower risk of heart attack, stroke, and certain cancers"

References

- The following references were deleted:
  - Hughes CP et al, 1982
  - Hachinski VC et al, 1975
  - Roman et al, 1993
  - Tatemichi TK et al, 1994
  - Brun A et al, 1994
  - McKeith IG et al, 1996

Appendix A: ADAPT field sites

"SEA - University of Washington" and "TAM - University of South Florida" were added

Appendix B: Design summary (same changes as outlined above)

Participant Consent Statement for Enrollment:

- "Some doctors have suggested that people taking celecoxib have an increased risk of a heart attack or stroke. There is a debate among doctors about whether this risk is real. The doctors who run ADAPT do not think the evidence for this risk is convincing. Since you may be assigned to take celecoxib, you should know of the current debate about its risks. If we learn more about these risks (or if we learn that they are not real risks) we will tell you about the new information on this topic." was added

Version 1.4 (19 Nov 2002)

The following changes were made to the exclusion criteria in section 3.3 and in appendix B:

- "Use of ≥4 doses per week of any of the following in the 28 days prior to the enrollment visit:
  - vitamin E (at doses > 600 IU per day)
  - non-aspirin NSAIDS **or** aspirin (>81 mg per day)
  - histamine H2 receptor antagonists
  - *Ginkgo biloba* extracts"

changed to

- "Use of ≥4 doses per week of either of the following in the 14 days prior to the enrollment visit:
  - non-aspirin NSAIDS **or** aspirin (>81 mg per day)
  - histamine H2 receptor antagonists"

- "History of hypersensitivity or anaphylactoid response to sulfa drugs or other NSAIDs (such as aspirin, ibuprofen, celecoxib, naproxen)"

changed to

"History of hypersensitivity or anaphylactoid response to sulfonamide antibiotics (e.g., Bactrim, Septra, Gantrisin, Gantanol, Urobak), or to aspirin or other NSAIDs (e.g., ibuprofen, diclofenac, celecoxib, naproxen)"

The following change was made to section 4.3:

"Serious adverse events must be reported to the Coordinating Center within 1 working day of the field site personnel learning of the event."

changed to:

"Serious adverse events thought to be possibly, probably, or definitely associated with use of study drug must be reported to the Coordinating Center within 1 working day of the field site personnel learning of the event."

The following changes have been made to the prototype consent statements in Appendix C:

- The five prototype consent statements have been revised to reflect the transfer of the Chairman's Office from the Johns Hopkins University to the University of Washington.
- The Risks/Discomforts section of the prototype ADAPT Participant Consent Statement for Enrollment has been revised as follows:

  "The study drugs can cause stomach irritation. This can range from mild stomach upset to ulcers. Very rarely, people with ulcers bleed from their stomachs. In older people who are **not** taking medicines like the study drugs, a bleeding ulcer occurs in about 5 people out of every 1000 in 1 year. Sometimes people have to be hospitalized for a bleeding ulcer. Most people recover, but a small number die from a bleeding ulcer. We do not think taking celecoxib will increase your risk of a bleeding ulcer much, if at all. Taking naproxen may increase your risk to about 10 in 1000. We will monitor you for any problems of this sort. If you develop any stomach problems, you will stop taking the study drug."

  changed to

  "The study drugs can cause irritation of the stomach or intestine. Most people with this problem have only mild symptoms. Rarely, the irritation can cause an ulcer or perforation (hole) in the stomach or intestine. In older people who are **not** taking medicines like the study drugs, serious problems like a bleeding ulcer or hole in the stomach or intestine occur at a yearly rate of 0.5%, (that is, in about 5 people out of every 1000.) Sometimes people have to be hospitalized for these problems. Most of these people recover fully, but a small number die. In the study's first 17 months, bleeding ulcers or perforation occurred in ADAPT participants at a yearly rate of 0.6%. Since bleeding from the stomach or intestine can cause a low blood count, we will check your blood count at least twice a year while you are in ADAPT. If you develop stomach or intestine problems, you will stop taking the study drug."

Source documents

The following document is partially excerpted and partially adapted from the following source documents:

- The grant application for this project
- Other protocols developed at the Johns Hopkins Center for Clinical Trials

Executive summary

An intervention that delays onset of Alzheimer's disease (AD) by several years would yield huge public health benefits. Several studies suggest that non-steroidal anti-inflammatory drugs (NSAIDs) may produce such a delay. NSAIDs may also attenuate progressive age-related cognitive decline (ARCD) when this condition represents a prodrome of AD. Both outcomes can be evaluated definitively only in randomized trials. Such trials can also examine attendant risks of long-term NSAID use in the moderate doses that appear to afford protection against AD and ARCD. Improved safety may be available with selective cyclooxygenase-2 (COX-2) inhibitors, but, owing to the newness of these compounds and the lack of relevant data, it is not known whether COX-2 inhibition offers the protective effect apparent with conventional NSAIDs.

The Alzheimer’s Disease Anti-inflammatory Prevention Trial is a randomized, placebo-controlled, multicenter trial designed to test the efficacy of a non-selective NSAID and a selective COX-2 inhibitor for the prevention of AD and attenuation of ARCD. The treatment regimens, which are tested in parallel, are: naproxen sodium (220 mg b.i.d.), celecoxib (200 mg b.i.d.), and placebo. The trial is designed to enroll 2,625 dementia-free participants who are age 70 or older and have a history of Alzheimer-like dementia in a first degree relative. Participants will be evaluated with structured, standardized methods of assessment and diagnosis. Conspicuous decline in periodic cognitive assessment test results will identify participants with suspected incident dementia, who will then undergo further assessment.

The proposed sample size presumes 7 years of followup, with estimates of incidence beginning at 2.5% for the first year and increasing by a factor of 1.1 per year. It also takes into account attrition through mortality and other causes, and treatment “drop-outs” and “drop-ins.” It should provide 80% power (2-tailed α=0.05) to detect a 30% reduction in either active treatment group.

Contents

Document distribution i

Document history ii

Source documents xiv

Executive summary xv

1. Background and significance 1
   1.1. Public health significance of Alzheimer's disease and age-related cognitive decline . . 1
   1.2. Potential for prevention of Alzheimer's disease 1
   1.3. Potential for attenuation of age-related cognitive decline 2
   1.4. Overview of prospects for the prevention of Alzheimer's disease 2
   1.5. Potential of non-steroidal anti-inflammatory drugs as neuroprotective agents 3
   1.6. Rationale for study treatments and doses 4

2. Objectives 7

3. Design 8
   3.1. Design features 8
   3.2. Sample size and power 8
   3.3. Eligibility criteria 9
   3.4. Randomization 10
   3.5. Masking 11

4. Treatment plan 12
   4.1. Treatment groups 12
   4.2. Compliance monitoring 12
   4.3. Management and reporting of side effects and adverse events 12
   4.4. Criteria for study treatment termination or interruption 13

5. Recruitment, screening, eligibility evaluation, and enrollment 14
   5.1. Overview of recruitment 14
   5.2. Overview of screening, eligibility evaluation and enrollment 14
   5.3. Screening 14
   5.4. Eligibility evaluation visit 14
   5.5. Enrollment visit 15

6. Followup 16
   6.1. Overview of followup visits 16
   6.2. Cognitive assessment visits 16
   6.3. Interval visits 17
   6.4. Telephone contacts 17
   6.5. Unscheduled followup visits and contacts 17
   6.6. Data collection schedule 19

7. Neuropsychological testing and dementia assessment 20
   7.1. Description of neuropsychological tests 20
   7.2. Diagnosis of dementia 24
   7.3. Followup of participants diagnosed with dementia 25
   7.4. Incidence of Alzheimer's disease 25
   7.5. Diagnostic Review Committee 26

8. Analysis plan 27
   8.1. General principles 27
   8.2. Analysis of design variable 28
   8.3. Analysis of other outcomes 28

9. Treatment effects monitoring 30

10. Quality assurance and performance monitoring 31
   10.1. Overview 31
   10.2. Certification of field sites 31
   10.3. Training of staff 31
   10.4. Certification of staff 31
   10.5. Performance monitoring 32
   10.6. Site visiting 32
   10.7. Error detection 33

11. Investigational new drug application 34

12. Protection of human subjects 35
   12.1. Monitoring of IRB approval process 35
   12.2. Consent process 35
   12.3. Risks and potential benefits to participants 36
   12.4. Safety monitoring 36
   12.5. Confidentiality of participant data 37

13. Biohazards 38

References 39

Appendices 47
Appendix A: ADAPT field sites 48
Appendix B: Design summary 49
Appendix C: ADAPT consent statements 52
Appendix D: Glossary of abbreviations and definitions 71

1. Background and significance

1.1. Public health significance of Alzheimer's disease and age-related cognitive decline

Dementia and cognitive decline are common, well-recognized public health problems of old age. Numerous studies now suggest that the prevalence of dementia after age 85 exceeds 30%[1]. Alzheimer's disease (AD) is by far the most common age-related dementia. It has recently been projected that the prevalence of AD will nearly quadruple to 8.94 million cases in the U.S. in 50 years[2]. Current annual U.S. expenditures on this dreaded illness are believed to exceed $90 billion[3]. The prevention of AD is thus a top public health priority.

The elderly are also prone to milder cognitive syndromes that have been grouped under such rubrics as “benign senescent forgetfulness” and “age-associated memory impairment.” In some instances these conditions portend more serious cognitive disorders, notably AD. Although called “mild,” these disorders are a major hindrance to the daily functioning of millions. Their reported occurrence varies widely with criteria for their identification, but their frequency increases with age[4]. Given the well known growth in the elderly population of the developed world, particularly the marked growth in the numbers of those aged 85 and older, these milder cognitive disorders may become comparable to dementia as a source of disability and a target for prevention.

1.2. Potential for prevention of Alzheimer's disease

Evidence is growing for the perspective that AD pathogenesis evolves chronically, with latent and prodromal phases. Fundamental to this perspective is the discovery of the importance of the *APOE* genetic locus that encodes the lipid transport protein apolipoprotein E[5]. For some time it has been known that genotype at *APOE* modifies risk of AD. It is now clear that *APOE* also influences cognitive ability, neural anatomy, and central nervous system metabolism in a preclinical or latent period that precedes AD dementia by many years. Thus, in cognitively normal elderly male dizygotic twin pairs who were discordant for the high-risk ε4 allele at *APOE*, the men with ε4 scored lower than their twin brothers on some cognitive tests[6]. Similarly, in a population-based study of individuals over age 70, non-demented subjects with ε4 showed greater cognitive decline over 4 years than did individuals without ε4[7]. Work by Ohm[8] suggested that Alzheimer-like neurofibrillary changes may begin 50 years before onset of dementia symptoms, and that this process is again influenced by genotype at *APOE*. MRI volumetric studies[9], including investigations of monozygous twin pairs[10], showed reduced hippocampal volumes in association with the ε4 allele. Finally, ε4 was associated with reduced parietal glucose metabolism (suggestive of decreased neural activity) in normal relatives of AD cases with mild memory complaints[11]. Altogether, these findings support the notion[12,13] that there are genotype-related latent processes in the brains of individuals who develop AD, and that these changes appear decades before onset of dementia. The existence of a prolonged latent phase in the AD process may make it possible to intervene in this phase and delay or prevent its progression to age-related cognitive decline (ARCD) and dementia.

1.3. Potential for attenuation of age-related cognitive decline

For decades there has been debate over whether, and under what circumstances, mild cognitive impairment may progress to AD. The foregoing notion of AD as an extended process with a long latent phase and a shorter prodrome suggests that longitudinal cognitive change may denote an incipient Alzheimer process. Thus, there is growing adherence to the notion that progressive (even if mild) cognitive decline may represent an end of a continuous and unimodal distribution of age-related change, with AD at its opposite extreme. Accordingly, severity of deficit or more restrictive criteria (*viz.*, those suggesting progression or other stigmata of early Alzheimer change) have been used to identify persons who are more likely to "convert" to clear-cut dementia within a short time of observation. Thus, Katzman[14] found that severity of deficit at baseline was a strong predictor of subsequent decline. Flicker[15] found that 72% of 32 subjects with "mild cognitive impairment" had declined significantly after 2 years. Rubin[16] followed clinic patients with "very mild senile dementia of the Alzheimer type" and found similar rates. O'Connor[17] studied a representative community sample and found that 12 out of 24 subjects with "minimal dementia" progressed to at least "mild dementia" after 2 years. In a large longitudinal study of the Cambridge (UK) population, one-third of subjects who meet criteria for mild dementia progress within 2 years to show moderate to severe dementia[18].

Several population studies have suggested that cognitive decline (in addition to dementia incidence) is related to the presence of *APOE* ε4 alleles. Henderson's[7] study of 638 subjects found trends associating ε4 dose with decline. Feskens et al.[19] showed a similar relationship ($p = 0.02$) among a somewhat older Dutch cohort. Hyman et al.[20] studied the Iowa EPESE cohort (1899 subjects) over 4 to 7 years. They found a significant but weak association ($p = 0.045$) between ε4 (and also ε2, which may be protective) and decline on a delayed recall task. The association of APOE and ARCD adds substantially to the argument that the latter may be a prodrome of AD.

1.4. Overview of prospects for the prevention of Alzheimer's disease

The foregoing findings suggest that AD and some instances of ARCD have common antecedents and, possibly, common mechanisms. They may thus be susceptible to common means of prevention. Several strategies are available for such efforts at prevention:

- **Identification and investigation of genetic markers:** The discovery of such markers for AD, and the ongoing search for new genes, have spurred efforts to uncover their mechanisms of action[21]. The discovery of these mechanisms could then lead to rational treatment and/or prevention strategies, but such strategies are not yet available for testing.

- **Identification of risk factors for intervention strategies:** Early epidemiologic research into environmental modification of AD risk focused principally on the identification of factors associated with increased occurrence of the disease. However, the factors discovered, such as increased age, imputed genes, and head injury[22], are not readily amenable to prevention strategies.

- **Interventions based on identification of environmental factors associated with decreased occurrence of AD:** A surprising result of the search for environmental modifiers of AD risk was the finding that several common exposures appeared to be associated with decreased occurrence of AD. These exposures include non-steroidal anti-inflammatory drugs (NSAIDs), hormone replacement therapy (in post-menopausal women), anti-oxidants (possibly including red wine), and histamine H2 receptor antagonist drugs[23]. A number of the above have been tested as treatments in patients with AD[24,25,26,27], and most have not proven effective. However, by the time AD is definitively diagnosed, its pathogenic processes may no longer be amenable to intervention. Therefore, despite its failure as a treatment, a given compound could still delay the latent stage mechanisms of AD, and thus prevent the emergence of dementia symptoms or ARCD. Because considerable observational evidence suggests that prevention may be possible using NSAIDs, this trial will test the prevention efficacy of two different NSAIDs.

1.5. Potential of non-steroidal anti-inflammatory drugs as neuroprotective agents

Over the past decade, growing empirical evidence has suggested that common anti-inflammatory treatments may inhibit the pathogenesis of AD. Rheumatoid arthritis patients (and, later, leprosy patients treated with the anti-inflammatory drug dapsone) were shown to have a reduced occurrence of AD[28,29]. Interest in possible inflammatory mechanisms in AD thus arose[30,31]. There are now at least 21 reports that consider whether sustained use of anti-inflammatory treatments delays or prevents onset of AD [28,32,33,34,35,36,37,38,39,40,41,42,43,44,45,46,47,48,49,50,51]. Several of these studies[41,46,48] were population-based, and one[50] used a prospective design. Two[34,45] were conducted using cases and controls who were genetically matched (twins or siblings). These latter studies were designed to investigate whether a reduced risk, if any, associated with NSAID use resulted specifically from a delay in the symptomatic expression of a process that was otherwise genetically determined. Despite their variety in methodological approach, these studies have mostly converged in suggesting an inverse relationship between sustained prior use of anti-inflammatory compounds (especially NSAIDs) and risk of AD. The strength of the findings overall is suggested in a meta-analysis[52].

The above studies that report use of particular NSAIDs[34,50] note that ibuprofen (the most commonly used NSAID) is also the most frequently reported drug among those with NSAID exposure, while naproxen is second in frequency. Among other notable points, those reports that mention dose and duration of exposure suggest that odds ratios (ORs) of about 0.5 do not require extended periods of exposure (although many exposed subjects surely have this) or large doses of NSAIDs.

The most recently published observations on the neuroprotective prospective of NSAIDs come from the Cache County Study[53,54], which enrolled over 5,000 (90%) of the county’s eligible elderly population. The study’s baseline cognitive screening interview included an extensive questionnaire covering prospective AD risk or protective factors, with emphasis on medical and pharmacologic exposures, and a medicine chest inventory of all drugs in current use. The study then examined the association of NSAID use and prevalent AD, adjusting for age, sex, education, and number of APOE

ε4 alleles. Odds ratios were examined simultaneously for other medication groups including aspirin and non-aspirin pain relievers (mainly acetaminophen), as well as H2 receptor antagonists and other treatments for dyspepsia and acid-peptic disease. The following odds ratio estimates (with 95% confidence intervals) and p-values were obtained:

NSAIDs (no ASA, no H2Bs)	OR = 0.43 (0.23 - 0.75), p = 0.002
Aspirin (no NSAIDs, no H2Bs)	OR = 0.50 (0.34 - 0.73), p = 0.0003
NSAIDs+ASA (no H2Bs)	OR = 0.17 (0.04 - 0.48), p = 0.0002
≥ 2 NSAIDs (no H2Bs)	OR = 0.14 (0.02 - 0.45), p = 0.0002
APAP, etc. (no NSAIDs or ASA)	OR = 0.81 (0.55 - 1.18), p = 0.287

where ASA is aspirin (typically used in low dose for cardiovascular prophylaxis), "APAP, etc." is non-ASA, non-NSAID analgesics (mostly acetaminophen), and H2Bs are histamine H2 receptor antagonists. One interpretation of these findings is that sustained low or moderate (not "anti-inflammatory") doses of cyclooxygenase inhibitors can produce the apparent prevention effect.

All of the above observational data suffer from several potential difficulties that may produce misleading results. The most obvious of these is difficulty in accurate classification of exposures. Demented subjects cannot provide their own pharmacologic history information, and reliance on current medicine usage (from a medicine chest review) is likely to be a poor indicator of sustained long-term use prior to onset of illness. This problem is likely to create substantial "noise" in the data, the usual result of which will be to mask or weaken the appearance of a relationship of AD to prior drug use, if one exists. Other difficulties may produce biased or spurious results. Those with AD may be less likely to demand or comply with prescriptions for anti-inflammatory treatments. Physicians may be less likely to prescribe these treatments when their patients are frail or demented. Other, unsuspected sources of confounding may also lead to spurious findings. For these reasons, observational data can only be regarded as preliminary or suggestive. Proof of any protective or beneficial effect with NSAIDs (or other drugs) must derive from clinical trials.

1.6. Rationale for study treatments and doses

The non-aspirin NSAIDs were first introduced in the 1960s as safer and more potent alternatives to high dose aspirin treatment for arthritis and other inflammatory conditions. Subsequent research showed that their anti-inflammatory activity is due to the inhibition of prostaglandin synthesis. In the late 1980s it was discovered that there are two prostaglandin synthetase enzymes, which were termed cyclooxygenase-1 (COX-1) and cyclooxygenase-2 (COX-2)[55]. COX-1 is an important constitutive enzyme that is essential for a number of biological activities, such as the protection of the stomach lining from the tissue-destructive effects of gastric secretions. By contrast, COX-2 is usually produced in response to pro-inflammatory stimuli. All non-aspirin NSAIDs marketed prior to 1999 are inhibitors of both COX-1 and COX-2. Two NSAIDs, celecoxib and rofecoxib, which were approved by the U.S. Food and Drug Administration (FDA) in 1999 for treatment of arthritis and

inflammation, are COX-2-selective compounds. In the doses commonly used for anti-arthritic effects, the selective inhibitors cause fewer adverse events (in particular, fewer gastric and duodenal ulcerations and bleeds)[56].

In designing the Alzheimer's Disease Anti-inflammatory Prevention Trial (ADAPT), the investigators considered it important to test both a non-selective COX inhibitor and a selective COX-2 inhibitor. The safety of the selective COX-2 inhibitors makes them attractive for a primary prevention trial. However, none of the preliminary, observational data on the relationship of NSAID use and AD includes exposure to these compounds. Furthermore, both COX-1 and COX-2 have been observed repeatedly in brain[57]. Because there is no current consensus on the function of either, nor on their possible relationship to AD pathogenesis, it is not known whether inhibition of COX-1, COX-2, or both is necessary for the postulated protective action of NSAIDs. In addition, although the safety advantage of the selective compounds has been demonstrated at relatively high, anti-arthritic doses, it is not clear that the same advantage will obtain at lower doses, such as those commonly reported in the observational evidence on NSAIDs and AD. Finally, if effective and well tolerated, the low-cost, over-the-counter (dual-inhibitor) NSAIDs could allow for a more cost-effective public health intervention.

Naproxen sodium was chosen as the conventional NSAID because:

- Naproxen is a frequently reported exposure in observational studies on NSAIDs and risk of AD, although less commonly reported than ibuprofen. The limited data available do not suggest an advantage of one drug over the other, either for safety or for their neuroprotective potential.
- Naproxen is a relatively strong inhibitor of COX-1 and a moderate COX-2 inhibitor. It should thus be a good compound to compare with a selective COX-2 inhibitor.
- Like the COX-2 inhibitors (but unlike ibuprofen), naproxen has a relatively long half-life of about 12 hours and should therefore maintain good plasma and cerebrospinal fluid levels when given in a b.i.d. regimen. Also, compared to pharmacologically equivalent over-the-counter formulations of ibuprofen, naproxen requires administration of fewer pills.

Celecoxib was chosen as the selective COX-2 inhibitor for the following reasons:

- It was the first selective COX-2 inhibitor approved by the FDA, and it remains the agent with the most post-marketing safety surveillance data.
- The safety data suggest that celecoxib has a side effect profile that is at least as favorable as rofecoxib[56], and it may produce less difficulty with fluid retention as a result of inhibition of COX-2 in the distal renal tubule.

The dose of naproxen sodium to be used in the trial is 220 mg b.i.d., because this dose has significant safety advantages over larger doses. Also, this dose is thought to be in the equivalent

range of NSAID use that has appeared in epidemiological studies to be potentially neuroprotective. In the Baltimore Longitudinal Study of Aging, the median reported daily dose of ibuprofen (the most common NSAID exposure) was about 800 mg/day[58]. The approximate equivalent dose of naproxen sodium is 440 mg/day.

The trial's specified dose of celecoxib is 200 mg b.i.d., which appears to come close to achieving the full anti-inflammatory potential of this compound in humans while still maintaining a good safety profile. In clinical trials of celecoxib for treatment of rheumatoid arthritis, the 400 mg daily dose of celecoxib provided nearly the same efficacy as the 800 mg daily dose and little difference in safety from the 200 mg daily dose[56].

It is recognized that the doses of the trial’s two treatments are not pharmacologically equivalent (the celecoxib dose being somewhat stronger). The chosen doses allow for the potential of maximum effect while maintaining reasonable safety. In addition, if one or both treatments prove efficacious, the combination of treatments and doses allows inferences regarding how the prevention is achieved. That is, we may be able to determine whether COX-1 inhibition is necessary for prevention of AD and whether full anti-inflammatory or lower doses contribute to the treatment effect.

2. Objectives

Primary objective

- To evaluate the efficacy of naproxen sodium as compared to placebo, and of celecoxib as compared to placebo, for the prevention of AD

Secondary objectives

- To determine whether the study treatments can attenuate cognitive decline with aging
- To compare the safety of the study treatments with placebo, and with each other, regarding mortality and the occurrence of side effects

---

3. Design

3.1. Design features

The study is a randomized, multicenter clinical trial with three parallel treatment groups. Treatment assignment is stratified by field site and age category and employs a 1:1:1.5 assignment ratio among the three treatment groups. Treatment assignment is masked to participants and all field site personnel, including clinicians, neuropsychologists and psychometricians.

3.2. Sample size and power

Sample size is calculated to allow detection of at least a 30% reduction in the incidence of AD. Such a reduction will be detectable with 80% power and a two-sided $\alpha = 0.05$ over 7 years of followup. Calculations were based on the log-rank statistic, under the following additional parameter assumptions:

- **Incidence rate**: It is estimated that the incidence rate of this population, “enriched” for family history of Alzheimer-like dementia, will be 2.5% in the first year, and that there will be a 10% proportional increase in each of the subsequent years (2.75% in the second year, 3.03% in the third year, and so on).
- **Mortality rate**: The mortality rates projected for the study population represent a base mortality rate in the first year of 4%, increasing by a factor of 8.5% in the subsequent years (4.34% in the second year, 4.71% in the third year, and so on).
- **Losses to followup or refusals of further follow-up**: A loss of 5% of participants each year is assumed for reasons such as participant relocation or refusal to continue in the study.
- **Adherence to assigned treatment**: The power of the study is affected 1) when participants assigned to active treatment stop taking the study medication and do not thereafter take an NSAID on a regular basis and 2) when participants assigned to placebo become regular NSAID users. The first situation is projected to occur in 15% of participants assigned to active medication during the first year and in an additional 5% for each of the remaining years. The second scenario is thought to be less probable (participants will be more likely to become intermittent, rather than regular, users of NSAIDs). It is therefore estimated that 2.5% of participants assigned to placebo will become regular NSAID users in each year.

Under the assumptions outlined above, the required sample size is approximately 2,625.

3.3. Eligibility criteria

Inclusion criteria

- Age 70 years or older at time of the eligibility evaluation visit

- Family history of one or more first-degree relatives with Alzheimer-like dementia
- A collateral respondent available to provide information on the cognitive status of the participant and to assist with monitoring of trial medications, if needed

- Sufficient fluency in written and spoken English to participate in study visits and neuropsychological testing

- Willingness to limit use of the following for the duration of the study:
  - vitamin E (at doses > 600 IU per day)
  - non-aspirin NSAIDs **or** aspirin (> 81 mg per day)
  - histamine H2 receptor antagonists
  - *Ginkgo biloba* extracts

- Ability and intention to participate in regular study visits, in the opinion of the study physician

- Provision of informed consent

Exclusion criteria

- History of peptic ulcer disease complicated by perforation, hemorrhage or obstruction

- History of uncomplicated peptic ulcer with symptoms in the 28 days prior to the enrollment visit

- Clinically significant hypertension, anemia, liver disease or kidney disease according to guidelines provided in the ADAPT Handbook

- History of hypersensitivity or anaphylactoid response to sulfonamide antibiotics (e.g., Bactrim, Septra, Gantrisin, Gantanol, Urobak), or to aspirin or other NSAIDs (e.g., ibuprofen, diclofenac, celecoxib, naproxen)

- Concurrent use of warfarin, ticlopidine or any other type of anti-coagulant

- Concurrent use of systemic corticosteroids

- Use of ≥ 4 doses per week of either of the following in the 14 days prior to the enrollment visit:
  - non-aspirin NSAIDs **or** aspirin (> 81 mg per day)
  - histamine H2 receptor antagonists

- Current plasma creatinine ≥ 1.5 mg/dL

- Enrollment in any trial that is likely to interfere with ADAPT procedures or affect treatment outcomes

- Cognitive impairment or dementia according to criteria specified in the ADAPT Neuropsychology Manual

- Current alcohol abuse or dependence

- Any condition that, in the opinion of the study physician, makes it medically inappropriate or risky for the participant to enroll in the trial

3.4. Randomization

Randomization will be accomplished using an auditable, documented generation scheme that produces a reproducible order of assignment. The randomization schedules will be written by the Coordinating Center and designed to yield an expected assignment ratio of 1:1:1.5 for naproxen sodium, celecoxib, and placebo, respectively. The larger number in the placebo group allows for more precise estimation of the incidence of AD in this group, which will be the control group for both treatment comparisons. Randomization will be stratified by field site and by three age categories, and assignments will be generated in permuted blocks of 7 or 14 within these strata.

The randomization process for ADAPT is designed for remote administration on the field site data systems. The steps are:

- Field sites determine if candidate qualifies for enrollment through form-driven eligibility checks on completion of required procedures, collection of required data, conformance with eligibility criteria and provision of consent
- Field sites request a treatment assignment using the password-protected randomization program
- If eligibility and completion of enrollment procedures are confirmed by the randomization program, a treatment assignment is issued and the prospective participant is enrolled in the trial
- Treatment assignments will be masked to the participants and the field sites, but not to a restricted set of personnel at the Coordinating Center

- Treatment assignment is communicated via a medication bin identifier (see section 3.5)
- The field site computer generates a treatment assignment sheet and visit schedule for the participant with allowable time windows
- The field site personnel fax the treatment assignment sheet and date of initial followup visit to the Coordinating Center within 24 hours of generation

3.5. Masking

Masking of treatment will be accomplished via a double placebo design. Participants assigned to receive naproxen sodium will take both this drug and the placebo matched to celecoxib. Likewise, those assigned to receive celecoxib will take both this drug and the placebo matched to naproxen sodium. Those assigned to receive placebo will take the placebos matched to both drugs. The treatment assignment generated by the computer will be to one of 49 medication identifiers; both study drugs (naproxen sodium/matching placebo and celecoxib/matching placebo) will be specified, in the appropriate numbers and combinations, by these 49 medication identifiers. The drug distribution center will be responsible for bottling the study treatments and labeling the bottles with a medication bin identifier according to a code specified by the Coordinating Center.

With this bin system, unmasking of any one person assigned to a given bin automatically unmasks all other participants assigned to that bin. However, it is anticipated that the number of instances in which a participant's treatment assignment must be unmasked will be limited to unusual emergency situations such as accidental overdose. In all other circumstances, the treatment assignment will remain masked for a participant who is experiencing serious side effects. The participant will stop study drug until the symptoms are alleviated and until such time (if ever) that it is judged safe and appropriate to reinstate study treatment (see section 4.4).

4. Treatment plan

4.1. Treatment groups

Participants will be assigned to one of the following groups:

- Celecoxib (Celebrex®, Pharmacia), 200 mg b.i.d., given orally
- Naproxen sodium (Aleve®, Bayer), 220 mg b.i.d., given orally
- Placebo

4.2. Compliance monitoring

Compliance with assigned treatment will be monitored via participant interview at each visit or telephone contact, and via pill counts. Participants will be asked to return all study medicines and bottles (used and unused) at each study visit.

4.3. Management and reporting of side effects and adverse events

Management: At the enrollment visit and all subsequent visits, study personnel will review with participants the symptoms of known potential adverse effects of the study medications. Participants will be encouraged to report these and any other side effects.

All adverse events thought to be related to study treatment will receive prompt initial evaluation by a study physician at that site. Study personnel will be "on call" by beeper at all times. For the purpose of clinical management, participants should be assumed to be on active treatment. In consultation with the participant's physician, a decision will be made whether to interrupt the study medicines.

Reporting: Adverse events should be reported to the Institutional Review Board (IRB) of the field site at which they occur, according to that institution's requirements.

Non-serious events are to be reported to the Coordinating Center as part of regularly scheduled data collection. Serious adverse events thought to be possibly, probably, or definitely associated with use of study drug must be reported to the Coordinating Center within 1 working day of the field site personnel learning of the event. The Coordinating Center will compile and distribute safety reports to the field sites, Treatment Effects Monitoring Committee (TEMC), National Institute on Aging (NIA), the U.S. Food and Drug Administration (FDA), Bayer and Pharmacia, as appropriate. Detailed guidelines and procedures for evaluation and reporting of adverse events are in the ADAPT Handbook.

4.4. Criteria for study treatment termination or interruption

A participant's study treatment will be terminated for the remainder of the study if either of the following occurs:

- The participant develops serious complications of an ulcer, such as gastrointestinal bleeding, perforation, or obstruction

- Any condition that, in the opinion of the study physician, makes it medically inappropriate or risky for the participant to continue on study treatment

Study treatment will be interrupted under the following circumstances:

- If the participant develops any signs or symptoms suggestive of an ulcer or kidney disease, the participant is withdrawn from study treatment pending an evaluation by the study physician and primary care physician (plus a specialist if necessary). The participant is put back on study treatment at the discretion of the study physician.

- If the participant develops an elevated blood pressure, creatinine, or potassium or a decreased hematocrit, he or she is referred for evaluation and treatment. The study physician determines whether it is medically necessary to interrupt study treatment.

- If the participant requires systemic corticosteroids, or warfarin, ticlopidine or any type of anti-coagulant, study treatment is interrupted for the duration of usage.

- If the participant is taking $\geq$ 4 doses per week of any of the following, study treatment is interrupted:
    - vitamin E (at doses > 600 IU per day)
    - non-aspirin NSAIDs **or** aspirin (> 81 mg per day)

- The participant enrolls in any trial that is likely to interfere with ADAPT procedures or affect treatment outcomes

- If the participant develops any condition that in the opinion of the study physician makes it medically inappropriate or risky to continue treatment, study treatment is interrupted.

5. Recruitment, screening, eligibility evaluation, and enrollment

5.1. Overview of recruitment

The general approach to recruitment will include a mailing to Medicare beneficiaries, distribution of study brochures and fact sheets to local physicians and interest groups, planned speaking engagements at local retirement centers and community groups, and print and radio advertisements. In addition, each site director will develop a site-specific strategy based on the demographics and their knowledge of the local community.

5.2. Overview of screening, eligibility evaluation and enrollment

The enrollment process will involve a screening interview and two clinic visits. The first visit is the eligibility evaluation visit. The second visit is the enrollment visit.

5.3. Screening

At the initial contact (telephone or in person), study personnel will provide prospective participants with information about the study, and conduct a screening interview. Interviewers will obtain each person's age and family history and ask questions to identify persons with a family history of Alzheimer-like dementia. Respondents who do not fulfill age or family history criteria will be thanked and the interview terminated. Otherwise, interviewers will describe the important features of the study and other participation requirements. Prospective participants will be informed that they must identify a collateral respondent. A collateral respondent is defined as a spouse, adult child or other person familiar with the participant's health and daily functioning. Attendance by a collateral respondent is encouraged for all visits; however, it will be required for the eligibility evaluation visit. If the person is interested in participating, the interview will continue with a list of questions about the other specific eligibility requirements, and an eligibility evaluation visit will be scheduled.

5.4. Eligibility evaluation visit

At the eligibility evaluation visit study staff will:

- Discuss the consent for eligibility evaluation and ask the prospective participant to sign
- Discuss the consent for collateral respondent and ask the collateral respondent to sign
- Record medical history and review of systems
- Perform physical and neurological examinations
- Obtain name and address of personal physician, if any

- Record current use of proscribed and limited-use medications
- Administer the following neuropsychological tests (see section 7.1) to the prospective participant:
  - Modified Mini-Mental State Examination-Epidemiological
  - Hopkins Verbal Learning Test-Revised
- Ask the collateral respondent to rate the participant using the Dementia Severity Rating Scale
- Collect blood and urine for laboratory tests
- Discuss the consent for enrollment and the consent for DNA testing and banking with the prospective participant; the unsigned consent forms will be sent home with those who indicate their willingness to participate.

If entry criteria are satisfied, the prospective participant will be scheduled for an enrollment visit. Prospective participants whose cognitive status does not meet the study entry criteria may be referred for evaluation outside the study.

5.5. Enrollment visit

At the enrollment visit study staff will:

- Review eligibility
- Discuss the consent for enrollment and the consent for DNA testing and banking and ask participant to sign
- Review and record current medications
- Administer the following neuropsychological tests to the participant:
  - Modified Mini-Mental State Examination-Epidemiological
  - Digit Span
  - Generative Verbal Fluency
  - Narratives from the Rivermead Behavioral Memory Test
  - Hopkins Verbal Learning Test - Revised
  - Brief Visuospatial Memory Test - Revised
  - Self-rating of Memory Functions
  - Geriatric Depression Scale
- Ask the collateral respondent to rate the participant using the Dementia Severity Rating Scale
- Draw blood for APOE determination and DNA banking
- Obtain the randomized treatment assignment
- Dispense study medications and review instructions for medication use
- Review visit schedule, compliance monitoring, and adverse events reporting

6. Followup

6.1. Overview of followup visits

Followup will include both scheduled and unscheduled visits and contacts. The three types of scheduled visits and contacts are:

- Cognitive assessment visits (every 12 months after enrollment)
- Interval visits (1 month and 6 months after enrollment and every 12 months thereafter)
- Telephone contacts (3 months after enrollment and every 6 months thereafter)

Target dates for followup visits are calculated from the date of enrollment. The time intervals around visit target dates are referred to as visit windows. Visit windows are contiguous (i.e., there is always an open visit window, because as one visit window closes, the subsequent one opens). The closing of one visit window and the opening of another will occur at the midpoint between the target dates of two consecutive visits. Ideally, visits are to be scheduled within 2 weeks of the target dates.

The two types of unscheduled followup visits and contacts are:

- Participant initiated contacts
- Dementia evaluation visits

6.2. Cognitive assessment visits

Participants will be scheduled to return every 12 months after enrollment for a comprehensive followup visit. At the cognitive assessment visits study staff will:

- Review interval medical history
- Perform physical and neurological examinations
- Collect blood and urine for laboratory tests
- Review treatment compliance and adverse events
- Review and record current medications
- Receive and record the amount of unused study drug
- Dispense new supply of study drug
- Administer the following neuropsychological tests to the participant:
    - Modified Mini-Mental State Examination-Epidemiological
    - Digit Span
    - Generative Verbal Fluency
    - Narratives from the Rivermead Behavioral Memory Test
    - Hopkins Verbal Learning Test - Revised
    - Brief Visuospatial Memory Test - Revised
    - Self-rating of Memory Functions
    - Geriatric Depression Scale
- Ask the collateral respondent to rate the participant using the Dementia Severity Rating Scale

6.3. Interval visits

Participants will be scheduled to return at 1 month and 6 months after enrollment and every 12 months thereafter for a visit to monitor compliance and adverse events. At the interval visits study staff will:

- Review interval medical history
- Collect blood and urine for laboratory tests
- Review and record current medications
- Review compliance and adverse events
- Receive and record the amount of unused study drug
- Dispense new supply of study drug
- Refer participant to a study physician if participant exhibits a notable change in condition or is medically unstable

6.4. Telephone contacts

Participants will be contacted by telephone at 3 months after enrollment and every 6 months thereafter. The telephone interviewer will:

- Review interval medical history
- Review compliance and adverse events
- Review current medications

6.5. Unscheduled followup visits and contacts

In addition to the visits outlined in the above schedule, participants may also be asked to appear for other assessments as needed. Most often these additional visits will be to evaluate a new or altered medical condition. Other assessments will be scheduled specifically to evaluate an apparent decline in cognitive abilities.

Participant-initiated contacts

Participants may contact the field site personnel in the interim between scheduled visits regarding medical or cognitive problems that they are experiencing. Information will be recorded on the nature of the complaints and on any recommendation or referral made by field site personnel.

Dementia evaluation visits

Participants who meet criteria specified in the ADAPT Neuropsychology Manual will be referred for a dementia evaluation visit. At the dementia evaluation visit study staff will:

- Review interval medical history with participant and collateral respondent
- Conduct review of systems with participant and collateral respondent
- Conduct neurological examination with participant
- Conduct mental status examination with participant
- Review current medications with participant and collateral respondent
- Administer the following neuropsychological instruments to the collateral respondent:
  - Dementia Severity Rating Scale
  - Dementia Questionnaire - clinical revision
  - Neuropsychological Inventory
- Administer Dementia Evaluation Battery for participants
  - The CERAD Battery
    - Generative Verbal Fluency
    - Short Boston Naming Test
    - Mini-Mental State Examination
    - Word List Memory
    - Constructional Praxis
  - Trail Making Test
  - Logical Memory
  - Benton Visual Retention Test
  - Controlled Oral Word Association Test
  - Symbol Digit Modalities Test
  - Shipley Vocabulary
  - Self-rating of Memory Functions

Depending on the results of the dementia evaluation, participants may be referred for laboratory testing and neuroimaging. Guidelines for clinical interpretation of the battery are specified in the ADAPT Neuropsychology Manual, and procedures for further management of participants are outlined in the ADAPT Handbook.

6.6. Data collection schedule

	EL* visit	EN* visit	Followup contacts (months from EN)								
	-1	0	1	3	6	9	12	15	18	21	24...
**Type of visit**											
Eligibility evaluation visit	✓	.	.	.	.	.	.	.	.	.	.
Enrollment visit	.	✓	.	.	.	.	.	.	.	.	.
Cognitive assessment visit	.	.	.	.	.	.	✓	.	.	.	✓
Interval visit	.	.	✓	.	✓	.	.	.	✓	.	.
Telephone contact	.	.	.	✓	.	✓	.	✓	.	✓	.
**Procedures**											
Consent	✓	✓	.	.	.	.	.	.	.	.	.
Physical exam	✓	.	.	.	.	.	✓	.	.	.	✓
Medical history & review of systems	✓	.	✓	✓	✓	✓	✓	✓	✓	✓	✓
Neurological exam	✓	.	.	.	.	.	✓	.	.	.	✓
Laboratory tests	✓	.	✓	.	✓	.	✓	.	✓	.	✓
Collection of DNA and blood sample	.	✓	.	.	.	.	.	.	.	.	.
Review of compliance	.	.	✓	✓	✓	✓	✓	✓	✓	✓	✓
Review of medication use	✓	✓	✓	✓	✓	✓	✓	✓	✓	✓	✓
Review of adverse events	.	.	✓	✓	✓	✓	✓	✓	✓	✓	✓
Dispensing of study drug	.	✓	.	.	✓	.	✓	.	✓	.	✓
**Neuropsychological tests**											
Modified Mini-Mental State Examination-Epidemiological	✓	✓	.	.	.	.	✓	.	.	.	✓
Digit span	.	✓	.	.	.	.	✓	.	.	.	✓
Generative Verbal Fluency	.	✓	.	.	.	.	✓	.	.	.	✓
Narratives from Rivermead Behavioral Memory Test	.	✓	.	.	.	.	✓	.	.	.	✓
Hopkins Verbal Learning Test-Revised	✓	✓	.	.	.	.	✓	.	.	.	✓
Brief Visuospatial Memory Test - Revised	.	✓	.	.	.	.	✓	.	.	.	✓
Self-rating of Memory Functions	.	✓	.	.	.	.	✓	.	.	.	✓
Geriatric Depression Scale	.	✓	.	.	.	.	✓	.	.	.	✓
Dementia Severity Rating Scale	✓	✓	.	.	.	.	✓	.	.	.	✓

*EL = eligibility evaluation; EN = enrollment

7. Neuropsychological testing and dementia assessment

7.1. Description of neuropsychological tests

Overview

The eligibility evaluation battery is administered to prospective participants at the time of the eligibility evaluation visit. Two neuropsychological test batteries are used for participants who enroll in ADAPT. A cognitive assessment battery is administered to participants at cognitive assessment visits to monitor cognitive function over time. A separate dementia evaluation battery is administered to those participants who show features of questionable or probable dementia based on the cognitive assessment battery or on clinical judgment. The dementia evaluation battery is used as an aid in the identification and differential diagnosis of dementia.

Eligibility evaluation battery

The eligibility evaluation battery includes two paper-and-pencil cognitive tests and one questionnaire administered to the participant's collateral respondent. A trained and certified psychometrician administers each of the tests according to the guidelines provided in the Eligibility Evaluation Battery and the ADAPT Neuropsychology Manual.

Cognitive tests

- *Modified Mini-Mental State Examination - Epidemiological (3MS-E)*: The measurement of global cognitive function, including orientation, language, verbal recall, recognition, long-term memory, praxis, and constructional ability, relies mainly on an adaption of the 100-point 3MS-E[59]. The original test or one of the two equivalent alternate versions developed for the Cache County Study on Memory and Aging[53] is used.

- *Hopkins Verbal Learning Test - Revised (HVLT-R)*: One of the six equivalent alternate forms of the HVLT-R[60] is used to measure verbal learning and delayed recall. In this test, a list of twelve words - four from each of three semantic categories - is ready to participants. Three learning trials are followed, after 20 to 25 minutes, by a delayed recall trial.

Collateral respondent questionnaire

- *Dementia Severity Rating Scale (DSRS)*: The informant version of the DSRS[61] is administered to the collateral respondent to assess cognitive and functional impairment.

Cognitive assessment battery

The cognitive assessment battery includes six paper-and-pencil cognitive tests and two self-rating questionnaires administered to the participant, as well as one questionnaire administered to the participant's collateral respondent. A trained and certified psychometrician administers each of the tests according to the guidelines provided in the ADAPT Neuropsychology Manual.

Cognitive tests

- *Modified Mini-Mental State Examination - Epidemiological (3MS-E)*: The measurement of global cognitive function, including orientation, language, verbal recall, recognition, long-term memory, praxis, and constructional ability, relies mainly on an adaptation of the 100-point 3MS[59]. The original test or one of two equivalent alternate versions developed for the Cache County Study on Memory in Aging[53] is used.

- *Digit Span*: The Wechsler Adult Intelligence Scale – Revised Digit Span subtest[62] is used to assess auditory attention and working memory. Both forward and backward span is assessed. Both tests consist of six pairs of number sequences that the psychometrician reads aloud one at a time. After each sequence is read, the participant must repeat the digits back in the same (forward) or reverse (backward) order.

- *Generative Verbal Fluency*: This test measures verbal production, semantic memory, and language. Participants are asked to name as many supermarket items as possible in one minute.

- *Narratives from the Rivermead Behavioral Memory Test*: One of the four equivalent alternate versions of the Narratives from the Rivermead Behavioral Memory Test[63] is used to assess immediate and delayed verbal memory. A brief passage is read to the participant. Immediately following auditory presentation of the passage, the participant is asked to recall the story. A final delayed recall trial is administered 15-20 minutes later.

- *Hopkins Verbal Learning Test – Revised (HVLT-R)*: One of the six equivalent alternate forms of the HVLT-R[60] is used to measure verbal learning, recognition, and delayed recall. In this test, a list of twelve words—four from each of three semantic categories—is read to participants. Three learning trials are followed, after 20 to 25 minutes, by a delayed recall trial and then a yes/no recognition trial.

- *Brief Visuospatial Memory Test - Revised (BVMT-R)*: One of the six equivalent alternate forms of the BVMT-R[64] is used to assess immediate and delayed nonverbal memory. The participant is presented with a card depicting six simple designs. After the designs are studied for a brief time period, the card is removed and the participant must immediately draw the designs from memory. Approximately 10-15 minutes after initial presentation, the participant is asked to draw the designs again. Immediately thereafter, a yes/no recognition trial is administered.

Self-administered questionnaires

- *Self-Rating of Memory Functions*: To determine the participant's perception of memory and other cognitive changes, the 18-item Self-Rating of Memory Functions[65] is administered. Participants respond to 18 items that ask them to rate changes over the past year using a 9-point Likert scale. Each item asks participants to judge their memory now compared to an earlier time period.

- *Geriatric Depression Scale (GDS)*: To determine the participant's perception of depression, the 30-item GDS[66] is administered.

Collateral respondent questionnaire

- *Dementia Severity Rating Scale (DSRS)*: The informant version of the DSRS[61] is administered to the collateral respondent to assess cognitive and functional impairment.

Dementia evaluation battery

The dementia evaluation battery includes eleven paper-and-pencil cognitive tests and one memory rating scale administered to the participant, and three questionnaires administered to the collateral respondent. A trained and certified psychometrician will administer the tests according to the standardized guidelines provided in the ADAPT Neuropsychology Manual.

Cognitive tests

- *The CERAD Battery*: The battery developed by the Consortium to Establish a Registry for Alzheimer's Disease[67] is administered. This battery includes the following tests:

  Generative Verbal Fluency: This test measures verbal production, semantic memory, and language. Participants are asked to name as many animals as possible in one minute.

Short Boston Naming Test: A modification of the Boston Naming Test measures expressive language. Participants are asked to name 15 line drawings of varying difficulty.

Mini-Mental State Examination (MMSE): This 30-item brief cognitive test measures orientation, immediate and delayed memory, concentration, language and praxis.

Word List Memory: This test measures verbal learning and the ability to remember newly learned information. A list of ten printed words is read to the participant who is then asked to recall as many words as possible. This is followed by two additional learning trials. To assess delayed recall, participants are asked to recall the ten previously presented words after a period of approximately 5 minutes has elapsed from initial presentation. To measure recognition, the ten target words are presented among ten distracters.

Constructional Praxis: To assess visuomotor ability, four line drawings of varying complexity are presented to the participant for copying. After a time delay, participants are asked to recall and draw the four designs.

- *Trail Making Test*: This timed test is used to measure attention, executive function, and visuomotor tracking[68]. To complete Part A, the participant must draw lines to connect consecutively numbered circles. To complete Part B, they must connect consecutively numbered and lettered circles by alternating between the two sequences.

- *Logical Memory*: The Logical Memory I subscale of the Wechsler Memory Scale – Revised[69] is used to assess immediate verbal memory. Two brief passages are read to the participant. Immediately following auditory presentation of each passage, the participant is asked to recall the story. The Logical Memory II subscale of the Wechsler Memory Scale – Revised[69] is used to assess delayed verbal memory. Approximately 30 minutes after completion of Logical Memory I, participants are once again asked to recall the two verbal passages.

- *Benton Visual Retention Test (BVRT)*: One of three equivalent alternate forms of the BVRT[70] is used to assess immediate visual/nonverbal memory. In this test, a design is placed in front of the participant. After the drawing is studied for a brief time period, it is removed and the participant must immediately draw it from memory. Each form of the BVRT includes ten different designs of varying difficulty.

- *Controlled Oral Word Association Test (COWA)*: Fluency of speech is assessed using COWA[71]. The number of words produced within 1 minute for three specified letters of the alphabet is recorded.

- *Symbol Digit Modalities Test (SDMT)*: To measure complex scanning and visual tracking, the SDMT is used[72]. In this timed test, the digits 1 through 9 are associated with a nonsense symbol. When presented with a row of symbols and blank spaces, the participant must write the number in the space that corresponds to each symbol.

- *Shipley Vocabulary*: The vocabulary subtest of the Shipley Institute of Living Scale[73] is used to assess premorbid verbal ability or verbal IQ. The participant is asked to circle one of four words that most closely matches the meaning of a target word. The 40-item list is progressively more difficult.

Memory scale

- *Self-Rating of Memory Functions*: To determine the participant's perception of memory and other cognitive changes, the 18-item Self-Rating of Memory Functions[65] is administered. Participants respond to 18 items that ask them to rate changes over the past year using a 9-point Likert scale. Each item asks participants to judge their memory now compared to an earlier time period.

Collateral respondent questionnaires

- *Dementia Severity Rating Scale (DSRS)*: The informant version of the DSRS[61] is administered to the collateral respondent to assess cognitive and functional impairment.

- *Dementia Questionnaire (DQ-cr)*: The clinical revision of the Dementia Questionnaire[74] is administered to the collateral respondent to assess memory and activities of daily living. The DQ-cr is a 50-item semistructured inventory of dementia symptoms and medical history developed to aid in the differential diagnosis of dementia.

- *Neuropsychiatric Inventory (NPI)*: The NPI[75] is a structured informant interview to assess behavioral and psychiatric symptoms. Using a series of graded questions, the informant is asked whether the participant has experienced each of 10 domains in the past month: delusions, hallucinations, agitation, depression, anxiety, elation, apathy, disinhibition, irritability, and aberrant motor behavior

7.2. Diagnosis of dementia

The results of each dementia evaluation visit will be reviewed at a conference of the site clinical team. Ordinarily, this team will comprise the field site director, the associate field site director, the examining study physician and nurse, and the site neuropsychologist. The clinical team will record a judgment regarding the presence of dementia, as defined by DSM-IV criteria[76]. At this initial field site conference, one of the following three impressions will be reached:

- **Participant is thought to be cognitively normal**
  - Participant should remain on study treatment
  - Participant should return to regular followup and annual cognitive assessment

- **Participant meets the Petersen criteria for mild cognitive impairment (MCI) or appears to have a clinical syndrome consistent with prodromal AD**
  - Participant should remain on study treatment
  - Participant should return to regular followup and annual cognitive assessment
  - Field site staff should monitor for evidence of further decline that may warrant referral for a repeat DEV

- **Participant is thought to have a dementia syndrome**
  - Study personnel should refer participant for laboratory evaluation of thyroid stimulating hormone, rapid plasma reagin (for syphilis), and vitamin B_{12} and folate, as well as for standard clinical MRI (or, alternatively, CT scanning, if MRI is contraindicated); details regarding referral for laboratory tests and imaging are specified in the ADAPT Handbook
  - Upon completion of laboratory tests and imaging, a second field site conference should be scheduled to review the results and to record clinical impressions on the differential diagnosis of the presumed dementia syndrome; the field site clinical team should refer to section 9.2 of the ADAPT Handbook for specific instructions on diagnostic criteria
  - For the purposes of the followup procedures described in section 7.3, a participant should be considered to have a diagnosis of dementia only when that diagnosis is considered by the field site clinical team to be reasonably certain and irreversible.

7.3. Followup of participants diagnosed with dementia

When the field site clinical team has reached a reasonably certain diagnosis of irreversible dementia, they should direct the participant to stop study treatment and should refer him or her to a dementia clinic for care. For as long as possible, however, the participant should return for regular ADAPT followup and annual cognitive assessment. When individuals with dementia are considered unable to make decisions about their continued participation in the trial, consent for such participation must be obtained from a responsible third party, along with assent from the participant if this is meaningful.

7.4. Incidence of Alzheimer’s disease

For analyses of the incidence of AD, participants with a diagnosis of dementia will be considered to have the event when the field site team has reached a differential diagnosis of possible or probable AD using NINCDS/ADRDA criteria[77]. On occasion, at a subsequent visit participants with a prior diagnosis of possible or, rarely, probable AD will show a clinically unexpected change that calls the diagnosis into question. In such instances the site medical director or other study physician will schedule a new dementia evaluation visit, with followup laboratory testing if clinically indicated. However, even if a participant’s diagnosis is thought to have changed, for the purpose of

ADAPT and its outcome measures, the database diagnosis will not be revised and the participant remain off study medication. Such cases should receive particular attention by the Diagnostic Review Committee.

7.5. Diagnostic Review Committee

The Diagnostic Review Committee will comprise the field site directors or associate directors, the Study Chair, and at least one senior neuropsychologist. The Committee will meet approximately every 6 months to review all newly identified presumed and all substantially revised cases of dementia. Material to be reviewed include the results of the dementia evaluation battery, laboratory testing, and neuroimaging. The purpose of the Committee reviews is to promote the homogeneity and standardization of diagnoses across the ADAPT field sites.

8. Analysis plan

8.1. General principles

General principles for analysis include the following:

- The primary analysis will be performed according to participants' original treatment assignment (intention to treat), regardless of administered treatment
- All participants, including those who are found to be ineligible after randomization or those who withdraw from the study, will be counted in their assigned treatment group once that treatment assignment has been revealed
- All events following randomization will be counted
- Subjects with missing measures at a particular time will be excluded from analyses that require those measures but will not be excluded from other analyses for which data are available

Analyses will be done to look for differences in outcome between each of the groups receiving active treatment and the placebo-treated group. Results of analyses will be presented unadjusted and adjusted. Covariates to be used for adjusting treatment group effects will include field site (also a stratification variable), age at entry (a stratification variable in the form of age categories), APOE genotype, gender, and other prospective baseline risk factors chosen with clinical judgment and/or variable selection procedures such as forward selection. Exploratory analyses will be performed in which post randomization data, such as adherence to the assigned treatment regimen or treatment received, will be taken into account. In addition, treatment effects will be examined across various subgroups.

Formal adjustment procedures will not be used for p-values resulting from the comparisons of each of the active treatment groups with the placebo group or from multiple comparisons made during interim monitoring. In the comparisons of the active treatments to placebo, the two treatment comparisons (naproxen sodium vs. placebo and celecoxib vs. placebo) are viewed as tests of separate experimental hypotheses. With respect to interim monitoring, it is to be expected that numerous comparisons of treatment efficacy and safety must be performed over the course of a clinical trial. Rather than to adjust p-values for multiple comparisons, p-values will be interpreted as descriptive statistics of the evidence, and not as absolute indicators for a positive or negative result.

8.2. Analysis of design variable

The analysis of incidence of AD will be drawn from multivariate survival analytic models, which account for censoring and allow for adjustment for multiple covariates. In the principal analysis, time-to-event methods will be used to model the effect of assigned treatment on the development of AD. (Secondary analyses of the treatment effects will include adjustments for adherence to the assigned study treatment.)

The survival analysis models will control for age at entry and other prospective baseline risk factors. Because age is a strong determinate of risk for AD, and because the association of age and AD is not linear, various models that adjust for age may be created. At the least, age will be included in the same categories used for stratification at randomization (ages 70-74, 75-79, and 80+). Finer categories may be used if there is evidence of residual confounding from imbalance in the age distribution among the treatment groups. However, it is expected that with stratification and a planned sample size of 2,625, such confounding is unlikely. Age-by-treatment interactions also will be investigated using the above stratification categories.

Other covariates to be included in the survival analysis models will include a core set of established predictors of AD incidence, as determined from epidemiological studies. Such factors include *APOE* genotype, education, gender, use of estrogen, use of anti-oxidants, and a history of head injury (family history of dementia is irrelevant here, as it is a criterion for enrollment). Again, it is expected that these variables will be balanced among treatment groups at enrollment, but analyses will be done to assess whether observed treatment effects are independent of them. Other baseline variables may be included if they are unbalanced across treatment groups.

Proportional hazards models may fail to adequately describe the treatment effects. For example, such effects may become apparent only after a threshold amount of time in treatment with naproxen sodium or celecoxib. In other words, the treatment effect may depend on the duration of followup. Accordingly, interactions between the treatment effect and followup time, using time-dependent covariates for such interactions with treatment, may be considered.

8.3. Analysis of other outcomes

Other outcomes to be investigated include measures of cognitive function, mortality, and the occurrence of adverse events. Comparisons of cognitive function between treatment groups will employ longitudinal data analysis techniques (generalized estimating equation (GEE) regression) described by Liang and Zeger[78] that account for the within-subject correlation. This method generates robust estimates for the variance without relying on specific assumptions about the correlation structure. It also allows for the use of a treatment effect indicator along with adjustment for multiple confounders and treatment effect modifiers in the comparison of the patterns of cognitive decline in

the two treatment groups. The outcome measure in the regression model will be the rate of change from baseline in the cognitive screening scores, calculated at each time that the tests are administered. To protect against influence of outliers, the ranks of the rates of decline will be used for the determination of p-values.

Survival analysis methods will be used to compare mortality in each of the treatment groups to that in the placebo treated group. If mortality is differential by treatment group, further analysis will be done to determine the effect of the differential mortality on the primary analysis of AD incidence.

The frequency and nature of adverse events will be described in two ways. Descriptive analyses will summarize the occurrence in each of the treatment groups of adverse events noting their frequency, severity, and major organ system involvement. Another analysis will focus on prediction and explanation of adverse events using a composite outcome variable. That is, participants' cumulative history of adverse events during the trial will be classified as mild, moderate or severe. The frequency of these outcomes will be formally compared between the treatment groups. Chi-square statistics and related methods for ordinal categorical variables will be used. In addition, logistic regression models will be used to adjust the frequency of adverse events between the treatment groups for imbalances and baseline predictor variables (if any).

9. Treatment effects monitoring

The ADAPT Treatment Effects Monitoring Committee is responsible for reviewing the accumulating data related to safety and efficacy. The committee will meet at least twice per year and they may elect to have *ad hoc* meetings or teleconferences at their own initiative.

The committee comprises both voting and non-voting members. The voting members are not involved in the conduct of ADAPT and are free of affiliations with the manufacturers of naproxen sodium or celecoxib bearing on the trial. The non-voting members are representatives of the study leadership and will include persons who are involved in implementation of the protocol as well as those involved in data analysis.

Monitoring reports of the accumulating data presented to the TEMC will include treatment group comparisons of baseline characteristics, incidence of AD, changes over time in cognitive measures, mortality, and adverse events. The TEMC will not be masked to treatment assignment in their deliberations regarding safety and efficacy. Stopping guidelines may be used in the decision-making process, but no formal curtailment rules based on p-values will be employed. However, stochastic curtailment approaches (based on conditional power and type I errors) may be used. The TEMC, with the advice and consent of the Steering Committee, will decide upon the approaches used for decision-making.

The TEMC may recommend stopping the trial before its planned conclusion if they observe convincing evidence of a treatment difference in adverse events or AD incidence. However, it is not expected that the TEMC will recommend stopping the trial early because of attenuation of decline in cognitive measures alone (i.e., without simultaneous demonstration of efficacy in reducing incidence of AD).

Recommendations of the TEMC will be forwarded by the Chair of the TEMC to the Chair of ADAPT. The recommendations will be communicated from there to the Study Officers and the Steering Committee. In addition, a summary report will be provided to the study IRBs. The Steering Committee will review the TEMC's recommendations. It is anticipated that the Steering Committee will approve the recommendations and then communicate them to the appropriate entities. In the event that the Steering Committee disagrees with the TEMC recommendations, the Steering Committee will follow the procedures outlined in the ADAPT policy statement on treatment effects monitoring.

10. Quality assurance and performance monitoring

10.1. Overview

Quality assurance strategies for ADAPT include design strategies and monitoring activities. Design strategies include use of randomization to assign participants to treatment groups, masking data collectors to treatment assignment to the extent possible, requirement of certification of staff and sites, and formal training of staff in ADAPT procedures. Activities to monitor quality include performance monitoring, visits to field sites, and error detection procedures.

10.2. Certification of field sites

Study investigators will be required to complete a field site certification form that provides detailed information with regard to the space, facilities, and personnel at the site. One purpose of the form is to serve as a checklist for staff of the resources that need to be in place when participant activities begin. One of the items requested will be a copy of the IRB notice of approval for ADAPT and copies of the stamped ADAPT consent statements to be used at the site. The information provided will be reviewed by Coordinating Center staff prior to certification of a field site for data collection.

10.3. Training of staff

The Chairman's Office and Coordinating Center will train the physicians, nurses, coordinators, neuropsychologists and psychometricians in the standardized and uniform use of all assessment instruments prior to the randomization of participants in the trial. Training methods will include didactic instruction and clinical demonstrations. As appropriate, standardized methods for performing other study procedures will be outlined in the ADAPT Handbook and the ADAPT Neuropsychology Manual. Staff attending the training meetings will be responsible for training other staff at the field site in ADAPT activities.

10.4. Certification of staff

The purpose of the staff certification program is twofold. It identifies to the Coordinating Center and to the study group the staff who will collect and/or record certain items of data for ADAPT and who will make decisions relating to eligibility for ADAPT. Second, it makes the data collector aware that he/she is a part of ADAPT and has a responsible and identifiable role in the trial.

Functions for which ADAPT will certify staff include director, associate director, coordinator, data system operator, neuropsychologist, participant contact coordinator, screening interviewer, study nurse, study physician and study psychometrician. The listing of certified functions results from recognition that some data for the trial will be collected by ADAPT staff at the field sites, while other data may be collected under the ADAPT protocol but by staff not directly employed by ADAPT.

All certified staff will be required to read the consent forms, protocol and handbook, to complete a form identifying the functions for which they are applying for certification in ADAPT, and to complete a Knowledge Assessment form. They will be asked to sign a statement acknowledging that they have read the study materials; that they understand that ADAPT is a collaborative activity and that results will not be available until the trial is terminated; that they will adhere to high standards of integrity in the data collection, recording, and editing processes; and that they will treat all ADAPT data as privileged information and thereby protect the confidentiality of the study participants and the collaborative research team. Certification for neuropsychological testing and data entry will require achievement of a set standard of performance. Each staff member certified for one or more functions for ADAPT will be issued a personal identification number; this number will be recorded as required when completing data collection forms.

10.5. Performance monitoring

Performance monitoring will begin with the initiation of participant screening and will continue throughout the duration of the trial. Field sites will be monitored on a regular basis regarding the following:

- Rate of enrollment
- Protocol deviations
- Missed visits
- Losses to followup
- Completeness of data
- Percentage of data items requiring edit queries
- Percentage of discrepancies found in audited data items

Summaries of the above measures will be provided to the field sites and to NIA on a regular basis and to the TEMC whenever they meet. Review of performance data will be an agenda item for all Steering Committee meetings.

10.6. Site visiting

Site visits will be made to each of the field sites early in the course of recruitment and at regular intervals (probably yearly). The site visitors will review consent forms for enrolled participants, study documents, IRB approvals, scheduling and logistics, staffing, adverse events, protocol issues, forms management, data management, specimen shipment, and study drug accounting.

10.7. Error detection

The study will employ double data entry to reduce the occurrence of errors. In addition, extensive range and logic checks will be done during data entry, and these checks may be continually updated throughout the trial to address new data problems as they are discovered. Edit queries will be made to the field sites on a regular basis regarding inconsistencies that were not resolved at data entry. Periodically, additional batch edits related to consistency of data across forms and over time will be generated.

Periodic audits of subsets of the ADAPT database will be conducted, both through visits to the field sites and through a remote auditing procedure. At on-site visits, participant data will be chosen for verification from source documentation. For the remote auditing procedure, field sites periodically will be asked to send copies of the ADAPT participant form sets to the Coordinating Center, and the data on the forms will be checked against the database for discrepancies. The electronic log of attempted randomizations will be reviewed periodically at the Coordinating Center and field sites will be questioned about unusual occurrences.

11. Investigational new drug application

The trial is being conducted under Investigational New Drug (IND) number 60,739 which is held by the ADAPT Chair. Protocol amendments will be submitted to both the FDA and NIA. The study leadership is also responsible for meeting reporting regulations of the FDA with regard to adverse events and annual reports.

12. Protection of human subjects

12.1. Monitoring of IRB approval process

The IRB serving the Johns Hopkins University Bloomberg School of Public Health is considered the parent IRB for ADAPT. Prototype consent materials for ADAPT will be provided by the Coordinating Center. Field sites may add information and reformat information to conform with their local requirements, but deletion of information will not generally be permitted.

One of the requirements for certification of a field site to begin participant activities will be submission of the site's notice of IRB approval and a copy of each stamped consent form used at the site. These materials will be reviewed by Coordinating Center/Chairman's office staff for conformance with the prototype materials and deviations will be questioned as appropriate.

Field sites that have obtained IRB approval for a previous version of the protocol will inform their IRB of changes to the protocol. Protocol amendments and changes to the consent form will be distributed from the Coordinating Center via numbered memos. These amendments and changes will be submitted by the field sites to their IRB in writing. All adverse events thought to be related to study treatment and all serious adverse events, regardless of presumed relationship to the experimental treatment, will be reported to the parent IRB as well as to the field site IRBs.

12.2. Consent process

The consent process for ADAPT is perceived as a dialogue between participant and ADAPT staff, supported by discussions and written materials.

Prototype written materials include four separate consent forms for the participant and one consent form for the collateral respondent. The consent forms are designed to be introduced at specific points in the screening, enrollment, and followup process.

The consent for eligibility evaluation is signed at the first visit (eligibility evaluation visit). The participant is asked to sign this statement at the same visit at which he/she first sees the statement. Also at this visit, the collateral respondent is asked to sign a consent form.

In addition, the consent form for enrollment and the consent for genetic testing and DNA banking are reviewed with the participant at the eligibility evaluation visit. The participant is given unsigned copies of the consent forms to take home. The forms are then reviewed again with the participant and signed at the enrollment visit.

If at any visit a participant needs a referral for a dementia evaluation, the consent form for the cognitive testing and diagnostic evaluation will be discussed with the participant and his/her collateral respondent. The consent form will be signed at the time of the dementia evaluation visit.

12.3. Risks and potential benefits to participants

Risks: There are risks to participation in ADAPT for each treatment group. Like all participants, those assigned to placebo will be asked to limit the use of NSAIDs and other medications (see section 3.3). Thus, participants in this group may have an increased burden of pain or inflammation, for example.

Participants assigned to receive naproxen sodium or celecoxib may experience adverse effects of the study drug, such as water retention, increased blood pressure, stomach irritation, gastrointestinal bleeding, anemia, renal toxicity, or hepatotoxicity. The risk of serious adverse events is expected to be higher in the naproxen sodium group than in the celecoxib group.

The above risks are outlined in the Participant Consent Statement for Enrollment (see Appendix C). Participants are told that they may withdraw from the study at any time and that they may stop the study drug in order to take proscribed medications. Safety monitoring is described in section 12.4.

Potential benefits: If one or both of the active study treatments are found to be efficacious, participants taking the efficacious treatment(s) may benefit from delayed onset or prevention of AD. It also is possible that participants receiving naproxen could benefit from a lower risk of heart attack, stroke, and certain cancers, although these effects are still under investigation.

All participants may benefit from the regular, ongoing, medical monitoring and referrals (when indicated) that they will receive in ADAPT. Those who develop dementia while in the trial may benefit from early diagnosis, which could facilitate appropriate treatment and care. In addition, all participants may benefit from the satisfaction of contributing to medical knowledge.

Alternatives to participation: Because ADAPT is a primary prevention trial, there is no direct alternative to participation. Other primary prevention trials are in progress, but the geographical location of the participating sites for the most part does not coincide with that of the ADAPT sites. Because the study medications are available either over the counter (naproxen sodium) or by prescription (celecoxib), people interested in the prevention of AD could self-medicate. However, given the unknown safety and efficacy of such use, it is not an advisable alternative to study participation.

12.4. Safety monitoring

ADAPT study personnel will have frequent contact with participants both by phone and in person (see section 6). Hematology and serum chemistry analyses, urinalysis, and reviews of medical history and concomitant medications will be performed every 6 months. Physical and neurological examinations will be given every year.

Participants will be monitored regularly for signs or symptoms of adverse effects (see section 4.3). Criteria are provided in the protocol for termination and interruption of treatment (see section 4.4). In addition, the TEMC will regularly review and evaluate accumulating safety data (see section 9) and may recommend termination of one or all of the treatments if the risks become unacceptable.

12.5. Confidentiality of participant data

Field sites will be instructed to keep all participant data in a secure location. Names, social security numbers, addresses, and other such personal data will not be sent to the Coordinating Center. Data collected from study evaluations and interviews will be identified only by study ID codes, which will be the participant ID number and name code assigned at eligibility evaluation. Data without participant identifiers may be released to the FDA, NIA, or other regulatory groups for monitoring purposes without further written consent of the participant. Release of identifiable data to any other persons or organizations will require additional written consent of the participant.

13. Biohazards

Blood will be collected in ADAPT for hematology and serum chemistry analysis and for banking. Urine will be collected for urinalysis. In addition, at the enrollment visit, blood will be collected for APOE genotyping and for banking at a central laboratory. All personnel involved in collecting and handling biologic specimens are to follow appropriate precautionary procedures as currently recommended by the Centers for Disease Control and Prevention. Shipping of specimens are to be done in compliance with Federal regulations.

References

1 **Breteler** MMB, Claus JJ, van Duijn CM, Launer LJ, Hofman A. Epidemiology of Alzheimer's disease. *Epidemiologic Reviews* **1992**; 14:59-82.
(Breteler et al, 1992) **1**

2 **Brookmeyer** R, Gray S. Methods for projecting the incidence and prevalence of chronic diseases in aging populations: Applications to Alzheimer's disease. *Stat Med* **2000**; 19:1481-1493.
(Brookmeyer and Gray, 2000) **1**

3 **Ernst** RL, Hay JW. Economic research on Alzheimer disease: a review of the literature. *Alzheimer Disease and Associated Disorders* **1997**; 11:135-145.
(Ernst and Hay, 1997) **1**

4 **Larrabee** GJ, Crook TH, III. Estimated prevalence of age-associated memory impairment derived from standardized tests of memory function. *International Psychogeriatrics* **1994**; 6:95-104.
(Larrabee and Crook, 1994) **1**

5 **Plassman** BL, Breitner JCS. Apolipoprotein E and cognitive decline in Alzheimer's disease. *Neurology* **1996**; 47:317-320.
(Plassman and Breitner, 1996) **1**

6 **Reed** T, Carmelli D, Swan GE, et al. Lower cognitive performance in normal older adult male twins carrying the apolipoprotein E E4 allele. *Archives of Neurology* **1994**; 51:1189-1192.
(Reed et al, 1994) **1**

7 **Henderson** AS, Easteal S, Jorm AF, et al. Apolipoprotein E allele e4, dementia, and cognitive decline in a population sample. *Lancet* **1995**; 346:1387-1390.
(Henderson et al, 1995) **1, 2**

8 **Ohm** TG, Muller H, Braak H, Bohl J. Close-meshed prevalence rates of different stages as a tool to uncover the rate of Alzheimer's disease-related neurofibrillary changes. *Neuroscience* **1995**; 64:209-217.
(Ohm et al, 1995) **1**

9 **Soininen** H, Partanen K, Pitkanen A, et al. Decreased hippocampal volume asymmetry on MRIs in nondemented elderly subjects carrying the apolipoprotein E e4 allele. *Neurology* **1995**; 45:391-392.
(Soininen et al, 1995) **1**

10 **Plassman** BL, Welsh KA, Bigler ED, et al. Apolipoprotein E e4 and hippocampal volume in twins with normal cognition. *Neurology* **1997**; 48:985-989.
(Plassman et al, 1997) **1**

11 **Reiman** EM, Caselli RJ, Yun LS, et al. Preclinical evidence of Alzheimer's disease in persons homozygous for the e4 allele for apolipoprotein E. *New England Journal of Medicine* **1996**; 334:752-758.
(Reiman et al, 1996) **1**

12 **Breitner** JCS, Welsh KA. Genes and recent developments in the epidemiology of Alzheimer's disease and related dementia. *Epidemiologic Reviews* **1995**; 17:39-47.
(Breitner and Welsh, 1995) **1**

13 **Roses** AD. Apolipoprotein E alleles as risk factors in Alzheimer disease. *Annual Review of Medicine* **1996**; 47:387-400.
(Roses, 1996) **1**

14 **Katzman** R, Aronson M, Fuld P, et al. Development of dementing illnesses in an 80 year old volunteer cohort. *Annals of Neurology* **1989**; 25:317-324.
(Katzman et al, 1989) **2**

15 **Flicker** C, Ferris SH, Reisberg R. Mild cognitive impairment in the elderly: predictors of dementia. *Neurology* **1991**; 41:1006-1009.
(Flicker et al, 1991) **2**

16 **Rubin** EH, Morris JC, Grant EA, Vendena T. Very mild senile dementia of the Alzheimer type I. Clinical assessment. *Archives of Neurology* **1989**; 46:379-382.
(Rubin et al, 1989) **2**

17 **O'Connor** DW, Pollitt PA, Jones BJ, Hyde JB, Fellowes JL, Miller ND. Continued clinical validation of dementia diagnosed in the community using the Cambridge Mental Disorders of the Elderly Examination. *Acta Psychiatrica Scandinavica* **1991**; 83:41-45.
(O'Connor et al, 1991) **2**

18 **Brayne** C. Personal Communication.
(Brayne, 2000) **2**

19 **Feskens** EJM, Havekes LM, Kalmijn S, de Knijff P, Launer LJ, Kromhout D. Apolipoprotein e4 allele and cognitive decline in elderly men. *British Medical Journal* **1994**; 309:1202-1206.
(Feskens et al, 1994) **2**

20 **Hyman** BT, Gomez-Isla T, Briggs M, et al. Apolipoprotein E and cognitive change in an elderly population. *Annals of Neurology* **1996**; 40:55-66.
(Hyman et al, 1996) **2**

21 **Roses** A. Alzheimer's disease as a model of molecular gerontology. *Journal of NIH Research* **1995**; 7:51-57.
(Roses, 1995) **2**

22 **Katzman** R, Kawas C. The epidemiology of dementia and Alzheimer disease. In: Terry RD, Katzman R, Bick KL, eds. *Alzheimer Disease*. New York: Raven Press, Ltd., **1994**; 105-122.
(Katzman and Kawas, 1994) **2**

23 **Breitner** JC. The end of Alzheimer's disease? *Int J Geriatr Psychiatry* **1999**; 14:577-586.
(Breitner, 1999) **3**

24 **Rogers** J, Kirby LC, Hempelman SR, et al. Clinical trial of indomethacin in Alzheimer's disease. *Neurology* **1993**; 43:1609-11.
(Rogers et al, 1993a) **3**

25 **Scharf** S, Mander A, Ugoni A, Vajda F, Christophidis N. A double-blind, placebo-controlled trial of diclofenac/misoprostol in Alzheimer's disease. *Neurology* **1999**; 53:197-201.
(Scharf et al, 1999) **3**

26 **Mulnard** RA, Cotman CW, Kawas C, et al. Estrogen replacement therapy for treatment of mild to moderate Alzheimer disease: a randomized controlled trial. Alzheimer's Disease Cooperative Study. *JAMA* **2000**; 283:1007-15.
(Mulnard et al, 2000) **3**

27 **Sano** M, Ernesto C, Thomas RG, et al. A controlled trial of selegiline, alpha-tocopherol, or both as treatment for Alzheimer's disease. The Alzheimer's Disease Cooperative Study. *N Engl J Med* **1997**; 336:1216-22.
(Sano et al, 1997) **3**

28 **McGeer** PL, McGeer E, Rogers J, Sibley J. Anti-inflammatory drugs and Alzheimer disease. *Lancet* **1990**; 335:1037.
(McGeer et al, 1990) **3**

29 **McGeer** PL, Harada N, Kimura H, McGeer EG, Schulzer M. Prevalence of dementia amongst elderly Japanese with leprosy. Apparent effect of chronic drug therapy. *Dementia* **1992**; 3:146-149.
(McGeer et al, 1992) **3**

30 **Aisen** PS, Davis KL. Inflammatory mechanisms in Alzheimer's disease: implications for therapy. *Am J Psychiatry* **1994**; 151:1105-1113.
(Aisen and Davis, 1994) **3**

31 **Breitner** JCS. Inflammatory processes and anti-inflammatory drugs in Alzheimer's disease: a current appraisal. *Neurobiol of Aging* **1996**; 17:789-794.
(Breitner, 1996) **3**

32 **Rogers** J, Kirby LC, Hempelman SR, et al. Clinical trial of indomethacin in Alzheimer's disease. *Neurology* **1993**; 43:1609-1611.
(Rogers et al, 1993b) **3**

33 **McDowell** I, Hill G, Lindsay J, et al. The Canadian Study of Health and Aging: risk factors for Alzheimer's disease in Canada. *Neurology* **1994**; 44:2073-2080.
(McDowell et al, 1994) **3**

34 **Breitner** JCS, Welsh KA, Helms MJ, et al. Delayed onset of Alzheimer's disease with non-steroidal anti-inflammatory and histamine H2 blocking drugs. *Neurobiology of Aging* **1995**; 16:523-530.
(Breitner et al, 1995) **3**

35 **McGeer** PL, McGeer EG, Rogers J, Sibley J. Does anti-inflammatory treatment protect against Alzheimer's disease? In: Khachaturian ZS, Blass JP, eds. *Alzheimer's Disease: New Treatment Strategies*. New York: Marcel Dekker,Inc., **1992**; 165-171.
(McGeer et al, 1992a) **3**

36 **Heyman** A, Wilkinson WE, Stafford JA, Helms MJ, Sigmon AH, Weinberg T. Alzheimer's disease: a study of epidemiological aspects. *Annals of Neurology* **1984**; 15(4):335-341.
(Heyman et al, 1984) **3**

37 **French** LR, Schuman LM, Mortimer JA, Hutton JT, Boatman RA, Christians B. A case control study of dementia of the Alzheimer type. *American Journal of Epidemiology* **1985**; 121:414-421.
(French et al, 1985) **3**

38 **Jenkinson** ML, Bliss MR, Brain AT, Scott DL. Rheumatoid arthritis and senile dementia of the Alzheimer's type. *British Journal of Rheumatology* **1989**; 28:86-88.
(Jenkinson et al, 1989) **3**

39 **Broe** GA, Henderson AS, Creasey H, et al. A case-control study of Alzheimer's disease in Australia. *Neurology* **1990**; 40:1698-1707.
(Broe et al, 1990) **3**

40 **Graves** AB, White E, Koepsell TD, et al. A case-control study of Alzheimer's disease. *Annals of Neurology* **1990**; 28:766-774.
(Graves et al, 1990) **3**

41 **Beard** CM, Kokmen E, Kurland LT. Rheumatoid arthritis and susceptibility to Alzheimer's disease. *Lancet* **1991**; 337:1426.
(Beard et al, 1991) **3**

42 **Breteler** MMB, van Duijn CM, Chandra V, et al. Medical history and the risk of Alzheimer's disease: a collaborative re-analysis of case-control studies. *International Journal of Epidemiology* **1991**; 20(suppl 2):S36-S42.
(Breteler et al, 1991) **3**

43 **Henderson** AS, Jorm AF, Korten AE, et al. Environmental risk factors for Alzheimer's disease: their relationship to age of onset and to familial or sporadic types. *Psychological Medicine* **1992**; 22:429-436.
(Henderson et al, 1992) **3**

44 **Li** G, Shen YC, Li YT, Chen CH, Zhau YW, Silverman JM. A case-control study of Alzheimer's disease in China. *Neurology* **1992**; 42:1481-1488.
(Li et al, 1992) **3**

45 **Breitner** JCS, Gau BA, Welsh KA, et al. Inverse association of anti-inflammatory treatments and Alzheimer's disease: initial results of a co-twin control study. *Neurology* **1994**; 44:227-232.
(Breitner et al, 1994) **3**

46 **Kukull** WA, Larson EB, Stergachis A, et al. Non-steroidal anti-inflammatory drug use and risk of Alzheimer's disease. *Neurology* **1994**; 44(suppl 2):A237.
(Kukull et al, 1994) **3**

47 **Myllykangas** R, Isomaki H. Alzheimer's disease and rheumatoid arthritis. *British Journal of Rheumatology* **1994**; 33:501-502.
(Myllykangas and Isomaki, 1994) **3**

48 **Andersen** K, Launer LJ, Ott A, Hoes AW, Breteler MMB, Hofman A. Do nonsteroidal anti-inflammatory drugs decrease the risk for Alzheimer's disease? The Rotterdam Study. *Neurology* **1995**; 45:1441-1445.
(Andersen et al, 1995) **3**

49 **Rich** JB, Rasmusson DX, Folstein MF, Carson KA, Kawas C, Brandt J. Nonsteroidal anti-inflammatory drugs in Alzheimer's disease. *Neurology* **1995**; 45:51-55.
(Rich et al, 1995) **3**

50 **Stewart** WF, Kawas C, Corrada M, Metter EJ. Risk of Alzheimer's disease and duration of NSAID use. *Neurology* **1997**; 48:626-632.
(Stewart et al, 1997) **3**

51 **Henderson** AS, Jorm AF, Christensen H, Jacomb PA, Korten AE. Aspirin, anti-inflammatory drugs and risk of dementia. *International Journal of Geriatric Psychiatry* **1997**; 12:926-30.
(Henderson et al, 1997) **3**

52 **McGeer** PL, Schulzer M, McGeer EG. Arthritis and anti-inflammatory agents as possible protective factors for Alzheimer's disease: a review of 17 epidemiologic studies. *Neurology* **1996**; 47:425-432.
(McGeer et al, 1996) **3**

53 **Breitner** JCS, Wyse BW, Anthony JC, et al. APOEe4 count predicts age when prevalence of Alzheimer's disease increases--then declines. The Cache County Study. *Neurology* **1999**; 53:321 - 331.
(Breitner et al, 1999) **3, 21**

54 **Anthony** JC, Breitner JC, Zandi PP, et al. Reduced prevalence of AD in users of NSAIDs and H2 receptor antagonists: the Cache County Study. *Neurology* **2000**; 54:2066-71.
(Anthony et al, 2000) **3**

55 **Vane** JR. Towards a better aspirin. *Nature* **1994**; 367:243-249.
(Vane, 1994) **4**

56 Celebrex product insert. From *Physician's Desk Reference*. Medical Economic Company, **2000**.
(Celebrex, 2000) **5, 6**

57 **O'Banion** MK. Cyclooxygenase-2: Molecular biology, pharmacology, and neurobiology. *Crit Rev Neurobiol* **1999**; 13:45-82.
(O'Banion, 1999) **5**

58 **Corrada** M. Personal Communication.
(Corrada, 2000) **6**

59 **Teng** EL, Chui HC. The Modified Mini-Mental State (3MS) examination. *Journal of Clinical Psychiatry* **1987**; 48:314-318.
(Teng and Chui, 1987) **21**

60 **Brandt** J. The Hopkins Verbal Learning Test: development of a new verbal memory test with six equivalent forms. *The Clinical Neuropsychologist* **1991**; 5:125-142.
(Brandt, 1991) 21

61 **Clark** CM, Ewbank DC. Performance of the Dementia Severity Rating Scale: a caregiver questionnaire for rating severity in Alzheimer disease. *Alzheimer Disease and Associated Disorders* **1996**; 10:31-39.
(Clark and Ewbank, 1996) **22, 24**

62 **Wechsler** D. *Wechsler Adult Intelligence Scale - Revised, Manual*. New York: Psychological Corporation, **1981**.
(Wechsler, 1981) **21**

63 **Wilson** B, Cockburn J, Baddeley A, Hiorns R. The development and validation of a test battery for detecting and monitoring everyday memory problems. *J Clin Exp Neuropsychol* **1989**; 11:855-70.
(Wilson et al, 1989) **21**

64 **Benedict** RH, Schrectlen D, Groninger L, Dobrashki M, Shpritz B. Revision of the Brief Visuospatial Memory tests: Studies of normal performance, reliability and validity. *Psychological Assessment* **1996**; 8:145-153.
(Benedict et al, 1996) **22**

65 **Squire** LR, Wetzel CD, Slater PC. Memory complaint after electroconvulsive therapy: assessment with a new self-rating instrument. *Biol Psychiatry* **1979**; 14:791-801.
(Squire et al, 1979) **22**

66 **Yesavage JA, Brink TL, Rose TL, Leirer VO**. Development and validation of a geriatric depression screening scale: A preliminary report. *J Psychiatric Res* **1983**; 17:37-49.
(Yesavage et al, 1983) **22**

67 **Morris** JC, Heyman A, Mohs RC, et al. The Consortium to Establish a Registry for Alzheimer's Disease (CERAD). Part I. Clinical and neuropsychological assessment of Alzheimer's disease. *Neurology* **1989**; 39:1159-65.
(Morris et al, 1989) **22**

68 **Reitan** RM. *Trail Making Test: Manual for Administration and Scoring*. Tucson: Reitan Neuropsychological Laboratory, **1986**.
(Reitan, 1986) **23**

69 **Wechsler** D. *Wechsler Memory Scale - Revised Manual*. San Antonio: Psychological Corporation, **1987**.
(Wechsler, 1987) **23**

70 **Benton** A. *Revised Visual Retention Test* - 4th Edition. New York: The Psychological Corporation, **1974**.
(Benton, 1974) **23**

71 **Benton** A, Hamsher K. *Multilingual Aphasia Examination*. Iowa City: University of Iowa Press, **1988**.
(Benton and Hamsher, 1988) **23**

72 **Smith** A. *Symbol Digit Modalities Test-Manual*. Los Angeles: Western Psychological Services, **1982**.
(Smith, 1982) **24**

73 **Zachary** RA. *Shipley Institute of Living Scale - Revised Manual*. Los Angeles: Western Psychological Services, **1991**.
(Zachary, 1991) **24**

74 **Silverman** JM, Breitner JCS, Mohs RC, Davis KL. Reliability of the family history method in genetic studies of Alzheimer's disease and related dementias. *American Journal of Psychiatry* **1986**; 143:1279-1282.
(Silverman et al, 1986) **24**

75 **Cummings** JL, Mega M, Gray K, Rosenberg-Thompson S, Carusi DA, Gornbein J. The Neuropsychiatric Inventory: comprehensive assessment of psychopathology in dementia. *Neurology* **1994**; 44:2308-2314.
(Cummings et al, 1994) **24**

76 **American Psychiatric Association**. *Diagnostic and statistical manual of mental disorders*. 4th ed. Revised. Washington, D.C.: American Psychiatric Association, **1994**.
(Am Psychiatric Ass, 1994) **24**

77 **McKhann** G, Drachman D, Folstein M, Katzman R, Price D, Stadlan EM. Clinical diagnosis of Alzheimer's disease: report of the NINCDS-ADRDA Work Group under the auspices of the Department of Health and Human Services Task force on Alzheimer's disease. *Neurology* **1984**; 34:939-944.
(McKhann et al, 1984) **37**

78 **Liang** KY, Zeger SL. Longitudinal data analysis using generalized linear models. *Biometrika* **1986**; 73:13-22.
(Liang and Zeger, 1986) **28**

Appendices

Appendix A: ADAPT field sites 48
Appendix B: Design summary 49
Appendix C: ADAPT consent statements 52
Appendix D: Glossary of abbreviations and definitions 71

Appendix A: ADAPT field sites

ADAPT ID	Institution	Director
**Field sites**		
BAL	The Johns Hopkins University<br>Baltimore, Maryland	Constantine Lyketsos, MD, MHS
BOS	Boston University<br>Boston, Massachusetts	Robert Green, MD
ROC	University of Rochester<br>Rochester, New York	Pierre Tariot, MD
SEA	University of Washington<br>Seattle, WA	Suzanne Craft, PhD
SUN	Sun Health Research Institute<br>Sun City, Arizona	Marwan Sabbagh, MD
TAM	University of South Florida<br>Tampa, FL	Michael Mullan, MD, PhD
**Resource centers**		
CO	Chairman's Office<br>The Johns Hopkins University<br>Baltimore, Maryland	John Breitner, MD, MPH
CC	Coordinating Center<br>The Johns Hopkins University<br>Baltimore, Maryland	Curtis Meinert, PhD
NIA	Project Office<br>National Institute of Aging<br>Bethesda, MD	Neil Buckholtz, PhD

Appendix B: Design summary

Title
- Alzheimer's Disease Anti-inflammatory Prevention Trial (ADAPT)

Objectives
- Primary objective
  - To evaluate the efficacy of naproxen sodium as compared to placebo, and of celecoxib as compared to placebo, for the prevention of AD
- Secondary objectives
  - To determine whether the study treatments can attenuate cognitive decline associated with aging
  - To compare the safety of the study treatments with placebo and with each other regarding mortality and the occurrence of side effects

Type of trial
- Randomized, multicenter, masked, placebo-controlled
- Three parallel treatment groups
- Sample size: 2,625

Treatments
- Celecoxib, 200 mg b.i.d.
- Naproxen sodium, 220 mg b.i.d.
- Placebo

Stratification
- Field site
- Age category (3)

Masking
- Double-masked: treatment assignment is masked to participants and all field site personnel, including clinicians, neuropsychologists and psychometricians
- Masked assessment of all outcomes
- Unmasked treatment effects monitoring

Inclusion criteria
- Age 70 years or older at time of the eligibility evaluation visit
- Family history of one or more first-degree relatives with Alzheimer-like dementia
- A collateral respondent available to provide information on the cognitive status of the participant and to assist with monitoring of trial medications, if needed
- Sufficient fluency in written and spoken English to participate in study visits and neuropsychological testing

- Willingness to limit use of the following for the duration of the study:
  - vitamin E (at doses > 600 IU per day)
  - non-aspirin NSAIDs **or** aspirin (> 81 mg per day)
  - histamine H2 receptor antagonists
  - *Ginkgo biloba* extracts (> 120 mg per day)
- Ability and intention to participate in regular study visits, in the opinion of the study physician
- Provision of informed consent

Exclusion criteria

- History of peptic ulcer disease complicated by perforation, hemorrhage or obstruction
- History of uncomplicated peptic ulcer with symptoms within 4 weeks prior to enrollment
- Clinically significant hypertension, anemia, liver disease or kidney disease according to guidelines provided in the ADAPT Handbook
- History of hypersensitivity or anaphylactoid response to sulfonamide antibiotics (e.g., Bactrim, Septra, Gantrisin, Gantanol, Urobak), or to aspirin or other NSAIDs (e.g., ibuprofen, diclofenac, celecoxib, naproxen)
- Concurrent use of warfarin, ticlopidine or any other type of anti-coagulant
- Concurrent use of systemic corticosteroids
- Use of $\geq$ 4 doses per week of either of the following in the 14 days prior to the enrollment visit:
  - non-aspirin NSAIDs **or** aspirin (> 81 mg per day)
  - histamine H2 receptor antagonists
- Current plasma creatinine $\geq$ 1.5 mg/dL
- Enrollment in any trial that is likely to interfere with ADAPT procedures or affect treatment outcomes
- Cognitive impairment or dementia according to criteria specified in the ADAPT Neuropsychology Manual
- Current alcohol abuse or dependence
- Any condition that, in the opinion of the study physician, makes it medically inappropriate or risky for the participant to enroll in the trial

Criteria for study treatment termination

- The participant develops serious complications of an ulcer, such as gastrointestinal bleeding, perforation, or obstruction
- Any condition that, in the opinion of the study physician, makes it medically inappropriate or risky for the participant to continue on study treatment

Criteria for study treatment interruption

- If the participant develops any signs or symptoms suggestive of an ulcer or kidney disease, the participant is withdrawn from study treatment pending an evaluation by the study physician and primary care physician (plus a specialist if necessary). The participant is put back on study treatment at the discretion of the study physician.

- If the participant develops an elevated blood pressure, creatinine, or potassium or a decreased hematocrit, he or she is referred for evaluation and treatment. The study physician determines whether it is medically necessary to interrupt study treatment.
- If the participant requires corticosteroids, or warfarin, ticlopidine or any type of anti-coagulant, study treatment is interrupted for the duration of usage.
- If the participant is taking $\geq$ 4 doses per week of any of the following, study treatment is interrupted:
  - vitamin E (at doses > 600 IU per day)
  - non-aspirin NSAIDs **or** aspirin (> 81 mg per day)
- The participant enrolls in any trial that is likely to interfere with ADAPT procedures or affect treatment outcomes
- If the participant develops any condition that in the opinion of the study physician makes it medically inappropriate or risky to continue treatment, study treatment is interrupted.

Duration of followup
- Up to 7 years

Data collection schedule
- Eligibility evaluation visit
- Enrollment visit
- Interval visits at 1 month and 6 months after enrollment and every 12 months thereafter
- Cognitive assessment visits every 12 months after enrollment
- Telephone contacts at 3 months after enrollment and every 6 months thereafter
- Participant initiated contacts, as needed
- Dementia evaluation visits, as needed

Outcomes
- Incidence of Alzheimer's disease
- Change in cognitive measures
- Mortality
- Adverse events

Appendix C: ADAPT consent statements

Participant Consent Statement for Eligibility Evaluation 53
Participant Consent Statement for Enrollment 56
Participant Consent Statement for Genetic Testing and DNA Banking 62
Collateral Respondent Consent Statement 65
Participant Consent Statement for Cognitive Testing and Diagnostic Evaluation 68

Alzheimer's Disease Anti-inflammatory Prevention Trial Participant Consent Statement for Eligibility Evaluation

---

Purpose

You are being asked to answer some questions and have some tests done. The purpose of these questions and tests is to see whether you are eligible to enroll in the Alzheimer's Disease Anti-inflammatory Prevention Trial (ADAPT). We are talking to you about ADAPT for two reasons. You are 70 years old or older, and you have a father, mother, sister, or brother who has or had dementia, senility or Alzheimer's disease.

ADAPT is a research study being done at the University of Washington, The Johns Hopkins University and (local site). It is funded by the National Institutes of Health. The purpose of the study is to test whether certain drugs can prevent Alzheimer's disease. As you may know, Alzheimer's disease is an illness that causes loss of memory and other abilities like language and thinking. The drugs being tested in ADAPT are naproxen (Aleve®) and celecoxib (Celebrex®). Naproxen is used to reduce fever and to treat pain and inflammation from ailments like arthritis. Celecoxib is a new drug for arthritis that works in a similar way but has fewer known side effects. We do not yet know whether either drug will be able to prevent Alzheimer's disease. Recent research suggests that they might, but this idea has not yet been tested. The study may last up to 7 years.

Procedures

To find out if you may enroll in ADAPT, we need to do the following:

- Ask you about your family medical history, your past and present health problems, and the medicines that you take
- Test your memory and thinking abilities; the tests will take about 25 minutes
- Take your blood pressure
- Do a physical exam and test your nervous system
- Collect a urine sample from you to run some tests
- Take 5 or 6 tablespoons of blood from a vein in your arm to run some tests

- Ask the person who came with you about your memory and daily activities

- Ask you for the name of your doctor and permission to contact him or her

After we get the results from the tests, we will know whether you may enroll in ADAPT. If you are eligible and still interested, we will ask you to come back for another visit. At that time, we will tell you more about the study, including its risks and possible benefits. Then you can decide if you want to enroll.

If your blood pressure is high, you will not be able to join ADAPT right away. We will talk to you about ways to treat your high blood pressure. If your blood pressure goes down, then you can join ADAPT. Also, if the results of the laboratory tests are unusual, we may ask you to repeat the tests on another day. This is so we can be sure whether you qualify for the study.

If today's tests show that you do not qualify for the study, we will contact you to let you know why.

Risks/Discomforts

There is a small risk from taking your blood. Sometimes, people feel a slight discomfort or even pain. Some people may feel faint for a few minutes. You might get a bruise on your arm after giving blood. The bruise should go away in a few days.

Today's tests may show that you are having some problems with your memory or thinking. If this occurs, we may refer you for evaluation outside the study.

Benefits

There is no direct benefit from today’s tests. However, they are a necessary step if you want to be in ADAPT.

Alternatives to Participation

Your agreement to answer questions and have tests done today is voluntary. You may choose to stop the questions or tests at anytime. Agreeing to today’s procedures does not mean that you are agreeing to be in ADAPT. Your choices will not affect the care you receive at this institution. There will be no penalty or loss of benefits to which you are entitled.

Confidentiality

We will make every effort to keep the information you give us confidential. We will not tell anyone without your permission that you are thinking about joining this study. ADAPT staff will use ID codes, not your name or social security number, when recording information about you and your test results. Study files are kept in a secure place. Only people who work on the study will have access to your data.

Questions and Concerns

Before you agree to be tested, make sure that you have answers to your questions. The study site director, Dr. _________, and the staff at ____[phone number]____ will answer any questions you have about this study, now or later.

If you believe that you have been hurt by being tested for this study or that you are not being treated fairly, you may contact the people named above. You also may contact the __[name of IRB and Institution]____ at ___[phone number]__ or The Johns Hopkins University's Office for Research Subjects at (410) 955-3193. The study site director or someone in the offices named above will answer your questions. If necessary, they will help you get medical care if you feel you have been hurt by the study. The University of Washington, The Johns Hopkins University, __[field site]__, and the Federal government do not have any program to provide compensation for injury or other bad effects that are not the fault of the investigators.

If you agree to answer questions and be tested for this study, please sign your name and write the date below.

____________________	____________________	____________
Participant signature	Date of signature	ADAPT ID No.
____________________	____________________	
Witness signature	Date of signature	
____________________	____________________	
Investigator signature	Date of signature	

Note: The signed consent form must be retained in the participant's file at the study site, and a copy must be given to the participant.

Alzheimer's Disease Anti-inflammatory Prevention Trial Participant Consent Statement for Enrollment

Purpose

You are being asked to enroll in the Alzheimer's Disease Anti-inflammatory Prevention Trial (ADAPT). ADAPT is a research study that is being done at the University of Washington, The Johns Hopkins University and (local site). It is funded by the National Institutes of Health. The purpose of this study is to test whether the daily use of the drugs naproxen or celecoxib can prevent Alzheimer's disease. As you may know, Alzheimer's disease is an illness that causes loss of memory and other abilities like language and thinking. Naproxen is used to reduce fever and to treat pain and inflammation from ailments like arthritis. Celecoxib is a new drug for arthritis that works in a similar way but has fewer known side effects. Naproxen is available over-the-counter as Aleve®. Celecoxib is available by prescription as Celebrex®. We do not yet know whether either drug will be able to prevent Alzheimer's disease. Recent research suggests that they might, but this idea has not yet been tested. The trial may last up to 7 years.

You have been asked to join ADAPT because you are 70 years old or older and you have a family member who has or had dementia, senility or Alzheimer's disease. You also have had some tests that show you do not have memory problems and that you may enroll in ADAPT.

Procedures

People who enroll in the study will be assigned to one of the following treatment combinations:

- naproxen and a placebo for celecoxib (group 1)
- celecoxib and a placebo for naproxen (group 2)
- a placebo for celecoxib and a placebo for naproxen (group 3)

A placebo is a pill that looks like the study medicines but has no active drug in it. You will be assigned to one of the above treatment groups, by a chance process like flipping a coin. Your chances of being in group 1 or group 2 are equal, about 28.5% each. Your chance of being in group 3 is about 43%. In other words, you have a better-than-equal chance of receiving one of the two active study drugs.

You will take two pills by mouth twice a day. You will not know which type of pills you are taking. Also, your doctor, and the study doctors and nurses, will not know which type of pills you are taking. We do this so that the study results won't be influenced by anyone's opinion about the treatments.

We will schedule a study visit with you about 1 month from now and about 6 months from now. After that, we will schedule visits with you every 6 months. We will contact you by telephone between the visits. There will be a total of 2 visits and 2 telephone calls each year. One of the visits each year will take about 2 hours. The other visit will take about 1 hour. The telephone contacts will take about 20 minutes each.

Each time you come for a visit, you need to bring your study pills with you. We will then give you a new supply of pills. Also, we will ask you about other medicines that you are taking. We will check that these are safe to take with the study drugs.

At all of the study visits:

- We will ask you questions about your health.
- We will ask you if you have had any problems with taking the study pills.
- We will ask you to give a urine sample. We also will take 5 or 6 tablespoons of blood from a vein in your arm. These samples will be tested to make sure that you are not developing any problems with your blood count, kidneys, or liver.
- If we have any concerns about your health or memory, you will see a study doctor.

In addition, at the longer study visits:

- A study doctor will do a physical and neurological exam.
- We will ask you questions to test your memory and thinking.
- We will interview your companion (the person who agreed to come with you to these visits). We will ask your companion whether your memory and daily functioning have changed.

Three months after each visit, someone from the study will call you on the phone to ask about your health. You will also be asked you if you have had any problems with taking the study pills. The staff person also will ask you what other medicines you are taking.

During the study, some people may develop memory loss that seems worse than expected from normal aging. If this happens to you, we will ask you for permission to run some extra tests. The purpose of these tests is to see whether you have signs of Alzheimer's disease, or other conditions.

Risks/Discomforts

We think the study drugs are safe at the doses given. But there is a possibility that they can cause several kinds of discomfort or illness. Most of these will go away if you stop the treatment. We will be checking on you every 3 months so that we can tell you to stop the study drug if needed to protect your health.

- One possible side effect of the study drugs is water retention. This is a concern if you develop heart disease while you are in ADAPT. If this happens you will stop taking the study drugs. Water retention almost always goes away when the study drugs are stopped.

- The study drugs can also cause a rise in blood pressure. Your blood pressure will be checked at every visit. If your blood pressure goes up too much, we will tell you to stop taking the study drugs until it comes down.

- The study drugs can cause irritation of the stomach or intestine. Most people with this problem have only mild symptoms. Rarely, the irritation can cause an ulcer or perforation (hole) in the stomach or intestine. In older people who are **not** taking medicines like the study drugs, serious problems like a bleeding ulcer or hole in the stomach or intestine occur at a yearly rate of 0.5%, (that is, in about 5 people out of every 1000.) Sometimes people have to be hospitalized for these problems. Most of these people recover fully, but a small number die. In the study's first 17 months, bleeding ulcers or perforation occurred in ADAPT participants at a yearly rate of 0.6%. Since bleeding from the stomach or intestine can cause a low blood count, we will check your blood count at least twice a year while you are in ADAPT. If you develop stomach or intestine problems, you will stop taking the study drug.

- There is also some risk of bleeding if you have surgery while you are taking the study medicine. For this reason, you should tell all of your doctors that you are in this study and that you might be on naproxen or celecoxib. If you are planning any kind of surgery, even minor surgery, you should give this information to your surgeon as well. Also, before the operation, tell the study doctor that you are going to have surgery. The surgeon and study doctor might decide that you should stop the study medicine for a time before the surgery.

- In rare cases, the study drugs cause liver or kidney problems in people who already have some liver or kidney damage. At each visit, your blood and urine will be tested to see if your liver and kidneys are working well. If you develop liver or kidney problems, you will stop taking the study drugs. These problems almost always improve when the drugs are stopped.

- Some doctors have suggested that people taking celecoxib have an increased risk of a heart attack or stroke. There is debate among doctors about whether this risk is real. The doctors who run ADAPT do not think the evidence for this risk is convincing. Since you may be assigned to take celecoxib, you should know of the current debate about its risks. If we learn more about these risks (or if we learn that they are not real risks) we will tell you about the new information on this topic.

If you develop any problems from taking the study medicine, we will help you get medical care.

You can bill Medicare and any other insurance you have for that care. However, you are responsible for paying any costs that are not covered by Medicare or other insurance.

While you are taking the study drugs, you should not take any medicines that are similar to them. Also, you should not take medicines that may cause side effects when taken with the study drugs. The study nurse will give you a list of the medicines you should not take. The study nurse and doctor also can talk with you and your personal doctor about substitute medicines that you can take. If your doctor decides that you need to take any of the medicines on the list, you will have to stop taking the study drugs. You may be able to take the study drugs again later if you stop the other medicines.

If you need to stop taking the study drugs for any reason, you should continue to come to the study visits for your tests and exams.

Benefits

- You will see a doctor or a nurse every 6 months. If your health changes, the study doctor will recommend that you see your doctor.

- You will have tests and exams to check you memory and thinking once a year.

- We don't know yet whether either naproxen or celecoxib will reduce the risk of Alzheimer's disease and memory loss. If they do, you might benefit from the treatment, depending on which treatment group you are in. When the study ends, we will tell you the results and tell you which treatment you received.

- Naproxen may reduce the risk of heart attack, stroke, and certain types of cancer. Studies are now being done to see if these benefits are real.

- Many people join studies like this because they want to contribute to medical research. If you are such a person, you may find that this is a benefit.

Alternatives to Participation

Your participation in ADAPT is voluntary. You may choose not to join this study. You also may withdraw from the study at any time. Your choice will not affect your medical care at this institution. There will be no penalty or loss of benefits to which you are otherwise entitled.

Rights and Responsibilities

All people who take part in this study have certain rights and responsibilities. Your rights include the following:

- You may choose not to join the study.

- You may withdraw from the study at any time and still get care at this institution. However, we will still want to find out how you are doing even if you withdraw. Knowing the status of all participants at the end of the trial is important.

- Study staff will answer any questions or discuss concerns you may have now or in the future.

- Staff also will inform you of any new findings during the course of ADAPT that might influence your willingness to continue in the study.

People who enroll in this study also have certain responsibilities. The success of the trial depends on participants coming to their study visits. Regular visits are important so that we can collect the data needed to compare the treatments. You will be asked to:

- Come for your study visits as scheduled.

- Work with study staff to complete the study procedures and to provide information about your health.

- Tell study staff about changes in your address and phone number.

If you know now that you will not be able to do these things, you should not join the study.

Confidentiality

We will make every effort to protect your privacy and keep your data confidential.

- We will use only a number and a 5-letter code to identify your study records. We will collect personal information (home and work addresses and phone numbers and the names of two friends or relatives). However, these will not be entered into the data files used for this study.

- The study data are kept in a secure place. Only people working on the study will have access to study data.

- The identity of all study participants is kept confidential. We will not identify any individual when we publish the results of the study.

- We will not give information about you to anyone outside the study without your written consent. However, the companies that make the study drugs or the FDA may review your medical record. This part of their duty to evaluate the treatments used.

- All requests for information about your participation in ADAPT from any doctors or medical staff will be handled through written reports and/or phone calls as needed. Information will be released only with your consent.

Who may profit

If the results of ADAPT show that the study drugs prevent memory loss, the companies that make the drugs may profit. Also, some of ADAPT's researchers and/or their institutions might benefit financially.

Questions and Concerns

Before you agree to enroll in ADAPT, make sure that you have answers to all your questions about the trial. The study site director, Dr. ________________ , and the staff at ____[phone number]___ will answer any questions you have about this study, now or later. If you believe that you have been hurt by being in this study or that you are not being treated fairly, you may contact the people named above. You also may contact the ________________[name of IRB and Institution]________________ at __[phone number]__ or The Johns Hopkins University's Office for Research Subjects at (410) 955-3193. The study site investigator or someone in the offices named above will answer your questions. If necessary, they will help you get medical care if you feel you have been hurt by the study. The University of Washington, The Johns Hopkins University, ____[field site]____, and the Federal government do not have any program to provide compensation to you for injury or other bad effects that are not the fault of the investigators.

If you agree to participate in this study, please sign your name and write the date below.

______________________	______________	__________
Participant signature	Date of signature	ADAPT ID No.
______________________	______________	
Witness signature	Date of signature	
______________________	______________	
Investigator signature	Date of signature	

Note: The signed consent form must be retained in the participant's file at the study site, and a copy must be given to the participant.

Alzheimer's Disease Anti-inflammatory Prevention Trial Participant Consent Statement for Genetic Testing and DNA Banking

---

Purpose

You have agreed to join the Alzheimer's Disease Anti-inflammatory Prevention Trial (ADAPT). This trial is being done at the University of Washington, The Johns Hopkins University and (local site). It is funded by the National Institutes of Health. The purpose of the study is to test whether the drugs naproxen (Aleve®) and celecoxib (Celebrex®) help prevent Alzheimer's disease.

You are now being asked to give a blood sample for genetic testing. The gene we want to look at in ADAPT is called APOE. We will test your blood to see which pair of three APOE genes you have. People with some forms of this gene are more likely to get Alzheimer's disease at an earlier age. We will use the knowledge about APOE genes when we test the treatments being used in ADAPT.

If you agree, we also will store some of your blood as DNA for future testing. DNA is the part of your body's cells that contains genetic information. Your DNA may be used to study Alzheimer's disease or other diseases, or to design and evaluate new genetic tests. If your DNA is used in the design of a commercial product or process, such as a new genetic test, you will not profit from it.

Procedures

A study nurse will take 5 or 6 tablespoons of blood from a vein in your arm.

Risks/Discomforts

There is some risk from taking your blood. Sometimes, people feel a slight discomfort or even pain. Some people may feel faint for a few minutes. You might get a bruise on your arm after giving blood. The bruise should go away in a few days.

Benefits

You will not benefit directly from the APOE test or from giving a DNA sample. The benefit is that you are contributing to research.

Alternatives to Participation

You have already decided to join ADAPT. Your choice about giving a blood sample is a separate decision and is voluntary. You do not have to give a blood sample for genetic testing. Also, you may give blood for APOE testing for ADAPT but choose not to have your DNA stored. If you choose now to give a DNA sample, you may later decide to have your DNA sample destroyed. Your choices will not at any time affect the care you will receive at this institution. There will be no penalty or loss of benefits to which you are entitled.

Confidentiality

We will make every effort to keep your records confidential, within the limits of the law. Our procedures are:

- Your DNA will be stored with a number code. This code will be used to link the sample to your other records in the study data files. Nothing in the study data files, including your DNA sample, will be linked to your name or other personal information.

- We will not release any information about individual genetic tests. We will not tell you, your study doctor or nurse, or anyone else the results of your tests, even if you ask us to. This is for your protection.

Questions and Concerns

Before you agree to give a blood sample, make sure that you have answers to your questions. The study site director, Dr. ______________________, and the staff at ___[phone number]___ will answer any questions you have about this study, now or later. If you believe that you have been hurt by being in this study or that you are not being treated fairly, you may contact the people named above. You also may contact the ______________[name of IRB and Institution]________________ at __[phone number]__ or The Johns Hopkins University's Office for Research Subjects at (410) 955-3193. The principal investigator or someone in the offices named above will answer your questions. If necessary, they will help you get medical care if you feel you have been hurt by the study. The University of Washington, The Johns Hopkins University, _____[field site]____ and the Federal government do not have any program to provide compensation to you for injury or other bad effects that are not the fault of the investigators.

Options to Choose

I agree to the following (check as many as you wish):

- ☐ APOE testing
- ☐ Storage of my DNA for studies of Alzheimer's disease
- ☐ Storage of my DNA for other medical research

Please sign your name and write the date below.

_________________________	_________________________	____________
Participant signature	Date of signature	ADAPT ID No.
_________________________	_________________________	
Witness signature	Date of signature	
_________________________	_________________________	
Investigator signature	Date of signature	

Note: The signed consent form must be retained in the participant’s file at the study site, and a copy must be given to the participant.

Alzheimer's Disease Anti-inflammatory Prevention Trial Collateral Respondent Consent Statement

---

Purpose

____________________ is thinking about joining the Alzheimer's Disease Anti-inflammatory Prevention Trial (ADAPT). ADAPT is a research study that is being done at the University of Washington, The Johns Hopkins University and (local site). It is funded by the National Institutes of Health. We are looking for people 70 years old or older who have a father, mother, sister, or brother who has or had dementia, senility, or Alzheimer's disease.

The purpose of the study is to test whether the daily use of the drugs naproxen (Aleve®) or celecoxib (Celebrex®) can prevent Alzheimer's disease. Naproxen is used to reduce fever and to treat pain and inflammation from ailments like arthritis. Celecoxib is a new arthritis drug that works in a similar way but has fewer known side effects. The study may last up to 7 years.

If your friend or relative joins ADAPT, he or she needs to have someone come along to some of the study visits. We need to be able to ask someone close to the person about his or her memory and daily functioning. We are asking you to help him or her "try out" for and possibly take part in this study.

Procedures

Today we will ask you some questions about your friend or relative's memory and daily functioning. If we find out from todays tests that he or she does not qualify for ADAPT, your commitment ends here. However, if the person does qualify, we will ask you both to come back for another visit. At this visit we will ask your friend or relative to sign up for the study. If he or she does, your role could continue for a few years. At the "sign-up" visit, we will again ask you questions about the person's memory and daily functioning. We will ask you to come along to study visits once each year after that to answer the same questions.

If your friend or relative has some memory loss either now or during the study, we will ask you both to come to a special visit. At this visit, he or she will have more memory tests and a neurological exam. We also will ask you to answer three sets of questions about the person's memory and daily functioning.

Risks/Discomforts

There are no risks to you from helping your friend or relative take part in ADAPT. However, the study will require some of your time. Also, some people may not like to answer questions about another person. If you are such a person, you may feel awkward in this role.

Benefits

Many people who take part in studies like this one do so because they like to contribute to medical research. If you are such a person, you may find this a benefit of taking part in ADAPT. You also will be helping your friend or relative do something that he or she wants to do. This person could benefit from the care or treatment he or she receives in ADAPT.

Alternatives to Participation

Your role in ADAPT is voluntary. You may choose not to take part in ADAPT. You also may withdraw from your role at any time. In this case, we will ask your friend or relative to find someone else to come along to the yearly study visits. Your choice will not affect the care that you or your friend or relative will receive at this institution. There will be no penalty or loss of benefits to which the person is entitled.

Confidentiality

We will make every effort to keep the information you give us confidential.

- We will not reveal your identity.
- We will not show or tell the information you give us to anyone else, including your friend or relative, without your permission.
- Study data are identified by study ID codes only.
- Study data are kept in a secure place. Only people working on the study will have access to the data.

Who may profit

If the results of ADAPT show that the study drugs prevent memory loss, the companies that make the drugs may profit. Also, some of ADAPT's researchers and/or their institutions might benefit financially.

Questions and Concerns

Before you agree to take part in ADAPT, make sure that you have answers to all your questions about the trial. The study site director, Dr. ______________________, and the staff at ___[phone number]___ will answer any questions you have about this study, now or later. If you believe that you have been hurt by taking part in this study or that you are not being treated fairly, you may contact the people named above. You also may contact the _______________[name of IRB and Institution]_________________ at __[phone number]__ or The Johns Hopkins University's Office for Research Subjects at (410) 955-3193. The study site director or someone in the offices named above will answer your questions. If necessary, they will help you get medical care if you feel you have been hurt by the study. The University of Washington, The Johns Hopkins University, _____[field site]___ and the Federal government do not have any program to provide compensation to you for injury or other bad effects that are not the fault of the investigators.

If you agree to participate in this study, please sign your name and write the date below.

________________________	________________	__________
Participant signature	Date of signature	ADAPT ID No.
________________________	________________	
Witness signature	Date of signature	
________________________	________________	
Investigator signature	Date of signature	

Note: The signed consent form must be retained in the participant's file at the study site, and a copy must be given to the participant.

Alzheimer's Disease Anti-inflammatory Prevention Trial Participant Consent Statement for Cognitive Testing and Diagnostic Evaluation

---

Purpose

As you know, you are taking part in the Alzheimer's Disease Anti-inflammatory Prevention Trial (ADAPT). This research study is being done at the University of Washington, The Johns Hopkins University and (local site). It is funded by the National Institutes of Health. The purpose of the study is to test whether the daily use of the drugs naproxen (Aleve®) and celecoxib (Celebrex®) can prevent Alzheimer's disease.

We have tested your memory and thinking as part of ADAPT. The test results show that you may be having some problems in these areas. We are asking you to complete some extra tests for a more thorough check of your memory and thinking. We also would like to talk with your companion (the person who comes with you to study visits). We will ask your companion about any changes in your memory and daily functioning.

We do not yet know whether the problems you are having are a cause for concern. These tests will help us find out. Some of the possible reasons people have problems with memory and thinking include normal aging, depression, stroke, and Alzheimer's disease.

After all the testing is done, we will get in touch with you to discuss the results. You then can decide whether you want us to give your test results to your doctor and/or companion.

Procedures

- A doctor will talk with you about your memory. He or she also will ask you to take some pencil-and-paper tests. These tests will tell us how well you can remember and concentrate. The interview and testing will take about 2.5 hours.

- A doctor will ask your companion about your recent medical history, your ability to do routine tasks, your emotional health, and any changes in your appetite or sleeping habits.

- Depending on the results of the interview and tests, we may ask you to have a brain scan called an MRI. The results of the scan will help us to know whether you have a problem, and if so, what is causing it.

- Depending on the results of the interview and tests, we may ask you to have some lab tests. We may need to take blood from your arm.

Risks/Discomforts

- The risk from taking the tests of your memory and thinking is minimal. However, we understand that you may be worried, and this may make you nervous during the tests. The study staff can talk with you about any worries you have.

- The risk from the MRI scan is minimal, although some people feel uneasy in the enclosed space of the scanner.

- Taking blood may cause some slight discomfort or even pain. Some people may feel faint for a few minutes. You might get a bruise on your arm after giving blood. The bruise should go away in a few days.

Benefits

Your test results may be useful to your doctor in planning your medical care. We will ask for your permission to give the test results to your doctor. If you want, we will send your doctor a letter that describes the results of your tests and their meaning.

Alternatives to Participation

Your decision to have further tests of your memory and thinking is voluntary. You may choose not to have the tests. You also may choose to stop the tests at any time. Your choices will not affect the care you will receive at this institution. There will be no penalty or loss of benefits to which you are entitled.

Confidentiality

We will make every effort to keep your test results confidential. Your test results will not have your name on it. They will have your study ID number only. Your test results will be kept with the rest of your study records, which are stored in a secure place. We will not release your test results to anyone unless we have your permission.

Questions and Concerns

Before you agree to be tested, make sure that you have answers to your questions. The study site director, Dr. ____________, and the staff at ___[phone number]___ will answer any questions you have about this study, now or later. If you believe that you have been hurt by being in this study or that you are not being treated fairly, you may contact the people named above. You also may contact the _______________[name of IRB and Institution]_________________ at __[phone number]__ or The Johns Hopkins University's Office for Research Subjects at (410) 955-3193. The principal investigator or the people in the offices named above will answer your questions. If necessary, they will help you get medical care if you feel you have been hurt by the study. The University of Washington, The Johns Hopkins University, ___[field site]____, and the Federal government do not have any program to provide compensation to you for injury or other bad effects that are not the fault of the investigators.

If you agree to participate in this study, please sign your name and write the date below.

______________________	________________	__________
Participant signature	Date of signature	ADAPT ID No.
______________________	________________	
Witness signature	Date of signature	
______________________	________________	
Investigator signature	Date of signature	

Note: The signed consent form must be retained in the participant's file at the study site, and a copy must be given to the participant.

Appendix D: Glossary of abbreviations and definitions

AD	-	Alzheimer's disease
ADAPT	-	Alzheimer's Disease Anti-inflammatory Prevention Trial
APOE	-	the genetic locus that encodes Apolipoprotein E
ARCD	-	age-related cognitive decline
ASA	-	Acetysalicylic acid
b.i.d.	-	twice per day
BVMT-R	-	Brief Visuospatial Memory Test - Revised
BVRT	-	Benton Visual Reproduction Test
CERAD	-	Consortium to Establish a Registry for Alzheimer's Disease
CHR	-	Committee on Human Research
COWA	-	Controlled Oral Word Association Test
COX-1	-	cyclooxygenase-1
COX-2	-	cyclooxygenase-2
dL	-	deciliter
DNA	-	Deoxyribonucleic acid
DQ-cr	-	Demetia Questionnaire
DSRS	-	Demetia Severity Rating Scale
FDA	-	Food and Drug Administration
GDS	-	Geriatric Depression Scale
GEE	-	generalized estimating equation regression
Hg	-	mercury
HVLT-R	-	Hopkins Verbal Learning Test - Revised
ID	-	identification
IND	-	Investigational New Drug
IU	-	international units
IRB	-	Institutional Review Board
L	-	liter
mEq	-	milliequivalent
mm	-	millimeter
mg	-	milligram
MMSE	-	Mini-Mental State Examination
MRI	-	Magnetic resonance imaging
3MS-E	-	Modified Mini-Mental State Examination - Epidemiological
NIA	-	National Institute on Aging
NIMH	-	National Institute of Mental Health
NINCDS-ADRDA	-	The National Institute of Neurological and Communicative Disorders and Stroke and the Alzheimer's Disease and Related Disorders Associations

NINDS-AIREN	-	The National Institute of Neurological Disorders and Stroke and the Association Internationale pour la Recherche et l' Enseignement en Neurosciences
NPI	-	Neuropsychiatric Inventory
NSAIDs	-	non-steroidal anti-inflammatory drugs
OR	-	odds ratio
SDMT	-	Symbol Digit Modalities Test
TEMC	-	Treatment Effects Monitoring Committee
